# *BcMF26a* and *BcMF26b* Are Duplicated Polygalacturonase Genes with Divergent Expression Patterns and Functions in Pollen Development and Pollen Tube Formation in *Brassica campestris*

**DOI:** 10.1371/journal.pone.0131173

**Published:** 2015-07-08

**Authors:** Meiling Lyu, Youjian Yu, Jingjing Jiang, Limin Song, Ying Liang, Zhiming Ma, Xingpeng Xiong, Jiashu Cao

**Affiliations:** 1 Laboratory of Cell & Molecular Biology, Institute of Vegetable Science, Zhejiang University, Hangzhou, China; 2 Zhejiang Provincial Key Laboratory of Horticultural Plant Integrative Biology, Zhejiang University, Hangzhou, China; 3 Department of Horticulture, College of Agriculture and Food Science, Zhejiang A & F University, Lin’an, China; 4 State Key Lab of Agrobiotechnology Shenzhen Base, Shenzhen Research Institute, The Chinese University of Hong Kong, Shenzhen, China; Wuhan University, CHINA

## Abstract

Polygalacturonase (PG) is one of the cell wall hydrolytic enzymes involving in pectin degradation. A comparison of two highly conserved duplicated PG genes, namely, *Brassica campestris Male Fertility 26a* (*BcMF26a*) and *BcMF26b*, revealed the different features of their expression patterns and functions. We found that these two genes were orthologous genes of *At4g33440*, and they originated from a chromosomal segmental duplication. Although structurally similar, their regulatory and intron sequences largely diverged. QRT-PCR analysis showed that the expression level of *BcMF26b *was higher than that of *BcMF26a* in almost all the tested organs and tissues in *Brassica campestris*. Promoter activity analysis showed that, at reproductive development stages, *BcMF26b* promoter was active in tapetum, pollen grains, and pistils, whereas *BcMF26a* promoter was only active in pistils. In the subcellular localization experiment, BcMF26a and BcMF26b proteins could be localized to the cell wall. When the two genes were co-inhibited, pollen intine was formed abnormally and pollen tubes could not grow or stretch. Moreover, the knockout mutants of *At4g33440* delayed the growth of pollen tubes. Therefore, *BcMF26a/b* can participate in the construction of pollen wall by modulating intine information and *BcMF26b* may play a major role in co-inhibiting transformed plants.

## Introduction

Pollen plays a significant role in plant sexual reproduction. Pollen insufficiency severely limits seed production. A unique structure of pollen grain is the pollen wall, which protects male gametophytes against environmental stresses and is essential for pollination [1]. A typical angiosperm pollen wall mainly contains two layers, namely, exine and intine [2]. The highly sculpted exine is mainly composed of sporopollenin released from the tapetum [2]. Similar to other plant cell walls, intine is mainly composed of pectin [1, 3]. The structure of the pollen tube wall is formed by a primary cell wall that is mainly composed of newly synthesized pectin at the tip [1].

Pectin is detected in primary and secondary cell walls of plant cells [4]. It makes up ~35% of the primary walls in angiosperm as a class of molecules defined by the presence of galacturonic acid [5]. Polygalacturonase (PG) is a cell wall hydrolytic enzyme that catalyze the random hydrolysis and disassembly of the 1,4-α-D-galactosiduronic linkages of pectin [6]. PG participates in various stages of plant development, such as seed germination, organ abscission, pod and anther dehiscence, pollen grain maturation, pollen tube growth, and xylem cell formation [6–8]. The cell separation events described above have been thought to be involved in pectin degradation by PG genes [9]. The expression levels of PG genes significantly differ in different organs and tissues. More than 50% of *Arabidopsis thaliana* PG genes are highly expressed in floral tissues [10]. Several PG genes have been cloned and proven to be specifically or highly expressed in pollen. Expression and functional assays have confirmed that *QRT2* and *QRT3* are two PG genes essential for pectin degradation to separate microspores in *A*. *thaliana* [11, 12]. *BcMF2* and *BcMF9* regulate the dynamic metabolism of pectin and subsequent intine formation in *Brassica campestris*, with *BcMF9* also participating in exine formation [13, 14]. Moreover, *BcMF6*, *BcMF16*, and *BcMF17* in *B*. *campestris* and *CpPG1* in zucchini (*Cucurbita pepo* L.) are important for pollen development [15–18].

PG belongs to a large gene family in plants. At least 67, 59, and 75 putative PG genes have been identified in *Arabidopsis*, rice (*Oryza sativa*), and *Populus* genomes, respectively [19–21]. These PG genes are divided into six clades (Clade A to Clade F) with differential expression patterns in different organs and tissues [22]. For example, one clade is expressed in vegetative tissues, whereas another is expressed in reproductive organs [23]. During the evolutionary process, PG duplicates undergone rapid expression divergence, and most of them have partial overlapping but distinct expression patterns in different tissues and organs [10]. Gene structure analysis has revealed that the functional divergence and differentiation of PG genes in land plants are partly attributed to intron losses [22]. In addition, changes in *cis*-regulatory modules of duplicated genes may lead to specific shifts in expression patterns between paralogs [24, 25]. The composition, expansion, and expression patterns of PG gene families have been characterized in different plants [10, 20, 26]. However, studies on expression, functional, and duplicated divergences of specific PG paralogs during evolution are rare.

In the current study, two duplicated PG genes, namely, *Bra011440* and *Bra037005*, in the *Brassica rapa* database, were cloned and characterized in *B*. *campestris* ssp. *chinensis* (syn. *B*. *rapa* ssp. *chinensis*). QRT-PCR analysis indicated that the expression levels of these two duplicated PG genes have significant divergences in homozygous male sterile plants ‘*Bcajh97-01A*’ and heterozygous male fertile plants ‘*Bcajh97-01B*’. Therefore, we renamed them as *Brassica campestris Male Fertility 26a* (*BcMF26a*) and *BcMF26b*. Moreover, we analyzed the divergences of their expression patterns by promoter activity analysis. The mutants of their homologous gene, *At4g33440*, in *A*. *thaliana* were also analyzed. Their functions in pollen development were investigated by the subcellular localization experiment, and amiRNA technology. The obtained results may be important for studying the evolution of duplicated genes and the functions of PG genes.

## Materials and Methods

### Plant materials and growth conditions

The *bcms* (*brassica campestris male sterility*) mutant of *B*. *campestris* L. ssp. *chinensis* Makino cv. Aijiaohuang named ‘*Bcajh97-01A/B*’, which was cultivated in the experimental farm of Zhejiang University, was used in this study. ‘*Bcajh97-01A*’ was homozygous male sterile plants and ‘*Bcajh97-01B*’ was heterozygous male fertile plant; the sibling (sister) lines segregated in a 1:1 ratio. Flower buds at different pollen development stages (Stage I, pollen mother cell stage; Stage II, tetrad stage; Stage III, uninucleate microspore stage; Stage IV, binucleate microspore stage; and Stage V, mature pollen stage) [27] were named as A1 to A5 in ‘*Bcajh97-01A*’ plants and B1 to B5 in ‘*Bcajh97-01B*’ plants. At flowering stage, flower buds from ‘*Bcajh97-01A*’ (A1–A5) and ‘*Bcajh97-01B*’ (B1–B5), five different organs (roots, stems, leaves, inflorescences, and germinal siliques) of ‘*Bcajh97-01B*’, and four floral parts (sepals, petals, stamens, and pistils) of B5 were collected and stored at −80°C.

*B*. *campestris* ssp. *chinensis* var. *parachinensis* is a subspecies of *B*. *campestris* L. ssp. *chinensis*, and a fast growing inbred with a growth period of only 50 d. This subspecies was used for our genetic transformation experiment. The transgenic plants were cultivated in a 22°C to 24°C growth chamber for phenotype observation.

The seeds of wild-type *Arabidopsis* (Col-0) and two *At4g33440* mutants (SALK_028430 and SALK_013967) were obtained from the ABRC database. The *A*. *thaliana* plants were grown under long-day conditions (16 h light/8 h dark) in a 22±1°C growth chamber for phenotype observation.

### DNA and RNA extraction, cDNA synthesis, and sequence amplification

Genomic DNA was isolated from leaves using the cetyltrimethylammonium bromide method [28]. Total RNA was purified from different tissues using TRIzol Reagent. The first strand cDNA was synthesized using a PrimerScript RT reagent Kit. The gene-specific primers (Primers 1 to 4, S1 Table) designed based on the sequence information of *BcMF26a* (gene ID: *Bra011440*) and *BcMF26b* (gene ID: *Bra037005*) in BRAD were used for homolog amplification of their DNA and cDNA sequences in *B*. *campestris*. The high fidelity thermostable DNA polymerase KOD was used for PCR. The PCR products were verified by sequencing.

### Gene structure and phylogenetic analysis

Gene structure was analyzed by FGENESH website. Sequence similarity alignment was carried out using ClustalX software. Molecular characteristics of the deduced protein were determined using ExPASy website. The signal sequence was analyzed with Signal P4.1 server. Collinear analysis was performed with GEvo website. Amino acid sequences in S2 Table from different plant species were retrieved from GenBank database or published references. The phylogenetic tree was generated with Mega6.0 software.

### QRT-PCR analysis

Two total RNAs were independently used for qRT-PCR to examine the transcript levels of *BcMF26a* and *BcMF26b* in *B*. *campestris*. The gene-specific primer pairs (Primers 5 and 6, S1 Table, S1 Fig) were designed based on the diverged sequences upstream of ‘ATG’, respectively, for independent expression examination of the two genes. The constitutively expressed *UBC-10* gene (Primer 7, S1 Table) was selected as an internal control. The CFX96 Real Time System machine and SYBR Premix Ex Taq Kit were used for the amplifications. The PCR products were sequenced to confirm the two genes. The 2^-ΔΔCt^ method was used to calculate the relative expression levels [29].

### Promoter activity analysis

The specific primers (Primers 8 and 9, S1 Table) were designed to isolate the promoter fragments of *BcMF26a* and *BcMF26b* based on their genome sequence information in BRAD. The putative promoter fragments included 50 bp to 150 bp downstream of ‘ATG’. The obtained putative promoter fragments were used to construct the fusion vectors pBGWFS7.0–proBcMF26a: GUS-GFP and pBGWFS7.0–proBcMF26b: GUS-GFP, with the destination binary vector pBGWFS7.0: GUS-GFP [30]. To examined the activity of the obtained promoter fragments, the two plasmids were transiently transformed into onion epidermal cells, respectively, by particle bombardment [31] using the Biolistic PDS-1000/He gene gun system. After 24 h of incubation, GFP-dependent fluorescence signals in the onion epidermal cells were observed by Fluorescent Microscope. Meanwhile, plasmids were introduced into *Agrobacterium tumefaciens* strain GV3101 and transformed into *Arabidopsis* wild-type plants using the floral dipping method [32] to produce transgenic GUS-expressing lines. The empty vector pBGWFS7.0: GUS-GFP was used as control.

The seedlings of T_1_ transgenic *Arabidopsis* plants were subjected to selection by spraying with 0.1% BASTA. The T_2_ transgenic lines were selected and used for histochemical GUS assay. The T_2_ seeds were grown on agar medium [consisting of half MS salts, 1.0% (w/v) sucrose, and 0.8% agar] for 4 d for seedling staining, as well as in soil for 35 d for inflorescence staining. Staining was performed as described by Sun et al. [33]. Subsequently, photographs of the seedlings and flower buds were taken using a stereomicroscope. Moreover, the floral buds at different development stages were dehydrated and embedded in Tissue-Tek^-^ OCT compound, and then frozen and sliced into 10μm sections. The sections were observed under Fluorescent Microscope. Populations derived from at least six independently transgenic lines were analyzed for each transformed construct.

### Subcellular localization of BcMF26a and BcMF26b proteins

The ORF fragments with specific restriction enzyme sites of *BcMF26a* and *BcMF26b* were amplified with gene-specific primer pairs (Primers 10 and 11, S1 Table). The resulting fragments were cloned into the pFGC: GFP vector to create the fusion vectors pFGC–BcMF26a: GFP and pFGC–BcMF26b: GFP. The two constructs were verified by sequencing and transformed into onion epidermal cells. The transformation process was consistent with the method of promoter activity analysis. Fluorescence signal was analyzed after 16 h of incubation. Moreover, the onion epidermal cell was plasmolyzed in 0.3 g mL^−1^ sucrose for 3 min and photographed by Fluorescent Microscope to investigate the GFP signal distribution.

### Artificial miRNA (amiRNA) construction and plant transformation

The amiRNA co-inhibiting *BcMF26a* and *BcMF26b* was designed as the procedure introduced in the MicroRNA Designer (WMD3) website according to the multiple target mode [34]. The amiRNA was constructed based on the miR164a backbone. The obtained amiRNA fragment was cloned into a modified pCAMBIA1301 vector, which was added with another CaMV35 promoter between the KpnI and SmaI restriction sites. The successful construct was transferred into *B*. *campestris* ssp. *chinensis* var. *parachinensis* though the *A*. *tumefaciens*-mediated transformation system described by Yu [35]. Meanwhile, empty vector pCAMBIA1301 was transformed as the negative control. Hygromycin was used to screen the transformed plants. With an identical primer (Primer 16, S1 Table), the mRNA levels of *BcMF26a* and *BcMF26b* in the transgenic lines were measured simultaneously by qRT-PCR analysis.

### Pollen analysis and pollen germination observation

The pollen grains from the dissected anthers of the flower buds about to open were collected and stained to analyze pollen viability. Alexander was used to assay the cytoplasm and cell wall [36]. 4', 6-Diamidino-2-phenylindole (DAPI) was used to stain the nuclei.

The samples for scanning electron microscopy (SEM), transmission electron microscopy (TEM), and semi-thin section observation were prepared using the procedure described by Huang [14]. The digital images of the pollen grains were obtained with a SEM. Semi-ultrathin sections (2 μm) were obtained, stained with dimethyl blue, and photographed with a Fluorescent Microscope. Ultrathin sections (50 nm) were stained with uranyl acetate and lead citrate, and then viewed in a TEM operating at 80 kV.

To observe pollen tube germination *in vitro*, pollen grains were collected and cultured as described by Jiang et al. [37]. After 4 h of incubation, the germinating percentage was calculated and the pollen tube morphologies were observed with Alexander staining under Fluorescent Microscope. Three independent transgenic lines and the corresponding control line were examined. More than six flowers buds from each plant were tested. At least 300 pollen grains were observed to calculate the average germination rate.

### Verification of T-DNA insertion mutants and *in vivo* pollen tube germination in *Arabidopsis*

Homozygous T-DNA insertion mutants of *At4g33440*, namely, SALK_013967 and SALK_028430, were identified using PCR with gene-specific primers (Primers 12 and 13, S1 Table). The coding region of *At4g33440* for RT-PCR was amplified using the specific-primer Primer 14 (S1 Table). *Tubulin* (Primer 15, S1 Table) was used as an internal control.

Preemasculated mature flowers were pollinated according to the following combinations: SALK_013967 × SALK_013967, wild-type × SALK_013967. Pistils were collected at 4, 12, and 24 h after hand-pollination, fixed with ethanol:acetic acid (v/v) = 3:1 solution, and softened with 8 M NaOH overnight. The pistils were washed in distilled water and stained with aniline blue solution in the dark for 3 h to 4 h. The stained pistils were photographed with Fluorescent Microscope under UV light. The same treatments were performed for the SALK_028430 mutant lines. Each combination was observed in triplicate.

## Results

### Sequence characterization analysis of *BcMF26a* and *BcMF26b*

**Fig 1:**
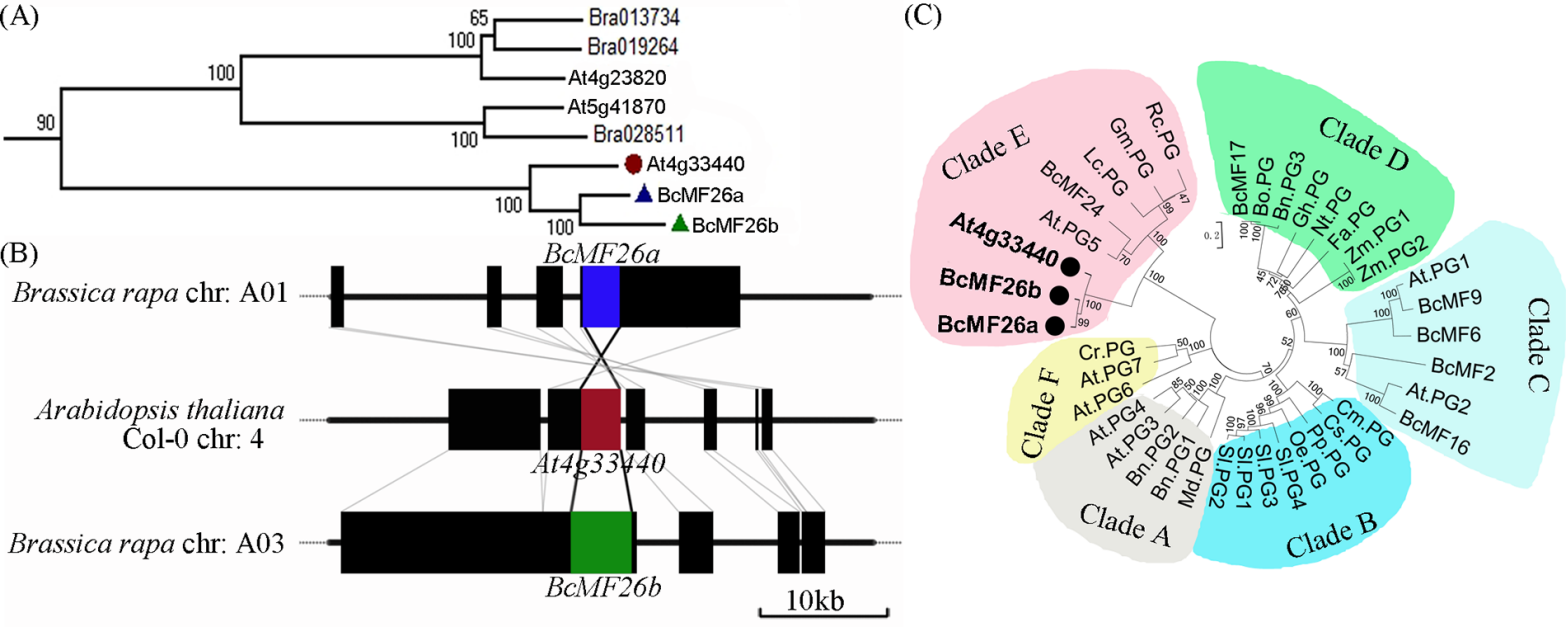
Sequence characterization and phylogenetic tree analysis. (A) The phylogenetic trees of PG genes from *Brassica campestris* and *Arabidopsis thaliana* genomes were generated using the neighbor-joining (NJ) method with 1000 bootstrap repeats (part of data displayed). *At4g33440*, *BcMF26a*, and *BcMF26b* are indicated by solid circle and triangles, respectively. (B) *BcMF26a*, *BcMF26b*, *At4g33440*, and their flanking regions representing ~45 kb of chromosomes are drawn to scale. Collinear conserved blocks are identified. The black solid lines indicate noncolinear chromosome fragments. The dotted lines represent the other regions of the chromosomes, which are not drawn to scale. The positions of *BcMF26a*, *BcMF26b*, and their orthologous gene *At4g33440* in *A*. *thaliana* are labeled with blue, red and green, respectively. Segmental chromosomal duplication and rearrangement are shown. (C) Phylogenetic tree constructed based on the amino acid sequence of *BcMF26a*, *BcMF26b*, *At4g33440*, and 35 PG genes from different plant species in S2 Table by NJ method. Confidence values from the bootstrap test (1000 replicates) are indicated by the numbers on the tree. The genes were clustered into six clades (Clade A to Clade F). *BcMF26a*, *BcMF26b*, and *At4g33440* are grouped in Clade E.

*BcMF26a* and *BcMF26b*, the orthologous genes of *At4g33440*, were found in BRAD using the basic local alignment search tool service. The phylogenetic tree analysis of PG genes from *A*. *thaliana* and *B*. *campestris* genomes also proved the high homology between *BcMF26a*, *BcMF26b*, and *At4g33440* (Fig 1A, part of data displayed). Collinear analysis was performed on *BcMF26a*, *BcMF26b*, and *At4g33440* (Fig 1B). *BcMF26a* was localized on Chromosome 1 (chr1) and *BcMF26b* was localized on Chromosome 3 (chr3) in the *B*. *campestris* genome. Meanwhile, *BcMF26a* and *BcMF26b* were aligned to the same locus, the PG gene *At4g33440*, in the *A*. *thaliana* genome. The regions immediately surrounding *BcMF26a* and *BcMF26b* on chr1 and chr3, respectively, exhibited the reverse orientation. The collinearly duplicated chromosomal blocks of the two genes were interspersed with chromosomal fragments of no apparent similarity. Moreover, the collinear conserved blocks between the two chromosomal regions preserved their orientations with respect to the target genes.

Gene sequences of *BcMF26a* and *BcMF26b* were isolated; both genes contained five exons and four introns (S1A Fig). The ORFs of *BcMF26a* and *BcMF26b* were 1425 and 1434 bp, encoding 474 and 477 amino acid residues, respectively. Their calculated molecular masses were 51.899 and 52.473 kDa, and their estimated pIs were 6.79 and 7.7. *BcMF26a* and *BcMF26b* had high similarities for ORFs and amino acid sequences (S1B and S1C Fig), which were 88.98% and 92.19%, respectively. However, the lengths of the four introns between the two genes were largely divergent (S2A to S2D Fig), and their sequence similarities were 71.88%, 62.64%, 68.35%, and 65.52% respectively. The lengths of the obtained promoter fragments were 2008 and 2059 bp for *BcMF26a* and *BcMF26b*, including 123 and 78 bp downstream of ‘ATG’, respectively. The sequences upstream of ‘ATG’ of the two promoter fragments showed significant divergences (S3 Fig), with a similarity of 63.20%.

Based on peptide sequence homology, PG genes from other plant species were found in the NCBI database by BLASTX. The phylogenetic tree was constructed using full-length amino acid sequences of *BcMF26a*, *BcMF26b*, and PG polypeptides from different plant species in S2 Table. The result revealed that *BcMF26a*, *BcMF26b*, and *At4g33440* were grouped into Clade E (Fig 1C). Furthermore, multiple sequence alignment with these PG proteins showed that they all contained three out of the four typical conserved domains of PG protein (S4 Fig).

### *BcMF26a* and *BcMF26b* had differential expression patterns

**Fig 2:**
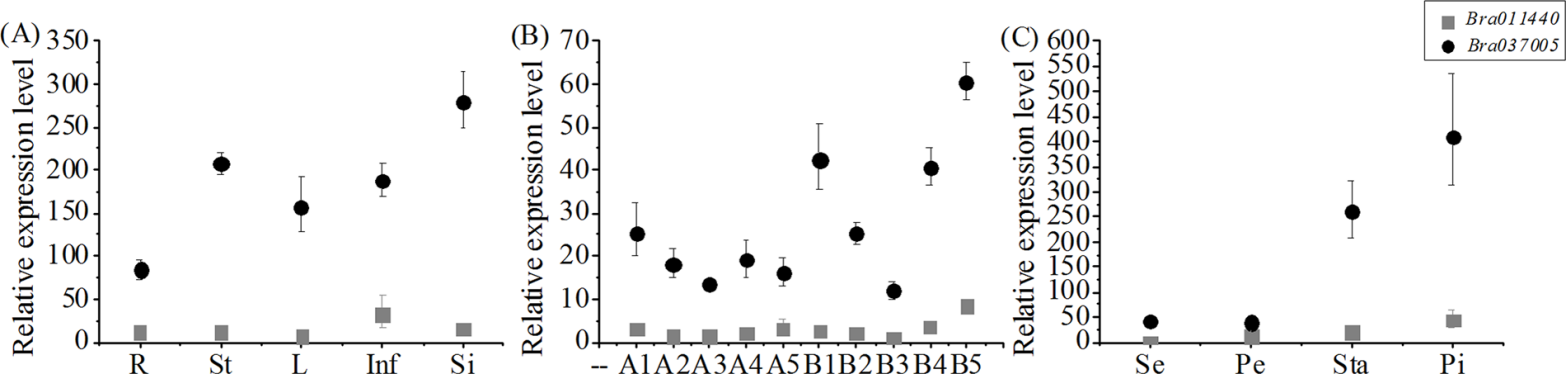
QRT-PCR analysis of *BcMF26a and BcMF26b* in different tissues and organs of *Brassica campestris*. (A) Relative expression patterns of *BcMF26a* and *BcMF26b* in roots (R), stems (St), leaves (L), inflorescences (Inf), and siliques (Si). Both *BcMF26a* and *BcMF26b* could express in all the five tested tissues. The transcript level of *BcMF26b* was higher than that of *BcMF26a*. (B) The relative expression levels of *BcMF26a* and *BcMF26b* in flower buds of ‘*Bcajh97-01A/B*’. The relative expression level of *BcMF26b* was much higher than that of *BcMF26a* in all the flower buds examined. The relative expression levels of both genes were much higher in buds of ‘*Bcajh97-01B*’ than those of ‘*Bcajh97-01A*’, except B3. A1–A5 and B1–B5 indicate flower buds at five developmental stages (Stage I to Stage V), namely, pollen mother cell stage, tetrad stage, uninucleate microspore stage, binucleate microspore stage, and mature pollen stage. (C) Relative expression levels of *BcMF26a* and *BcMF26b* in separate flower organs of B5, including sepals (Se), petals (Pe), stamens (Sta), and pistils (Pi). *BcMF26b* mainly expressed in stamens and pistils. The relative expression level of *BcMF26b* was much higher than that of *BcMF26a*. Standard errors for three independent experiments are shown.

The transcripts of *BcMF26a* and *BcMF26b* were detected in different organs and tissues of *B*. *campestris* using qRT-PCR analysis (Fig 2). In the five different organs of heterozygous male fertile ‘*Bcajh97-01B*’ plants, *BcMF26b* expression in stems, leaves, inflorescences, and siliques was 2.46, 1.87, 2.22 and 3.32 times higher than that in roots, respectively (Fig 2A). In the flower buds at five pollen developmental stages from the homozygous male sterile ‘*Bcajh97-01A*’ plants (A1–A5) and heterozygous male fertile ‘*Bcajh97-01B*’ plants (B1–B5) (Fig 2B), the relative expression levels of *BcMF26b* in B1, B2, B4, and B5 were higher than that in A1, A2, A4, and A5; the minimum expression level divergence was 1.38 times between B2 and A2. Moreover, the expression of *BcMF26b* in ‘*Bcajh97-01B*’ was much higher in B1, B2, B4, and B5, which were 3.59, 2.13, 3.45, and 5.13 times higher than that in B3, respectively. For the four floral parts of B5 (Fig 2C), *BcMF26b* was mainly expressed in stamens and pistils, with relative expression values of 259.57 and 409.20, respectively, but those in sepals and petals were only 40.69 and 39.48, respectively. *BcMF26a* was hardly expressed in all the tested organs and tissues compared with *BcMF26b* (Fig 2). The highest expression value was 43.11 in pistils, which was only 0.1 times that of *BcMF26b* in the same tissue. However, *BcMF26a* and *BcMF26b* had similar expression trends (Fig 2). The main expression divergences between *BcMF26a* and *BcMF26b* were the differences in expression levels in the tested organs and tissues.

‘*Bcajh97-01A*’ is a genetic male-sterile line that lacks mature pollen; ‘*Bcajh97-01B*’ is the fertile maintain line [38]. Given the different expression patterns in ‘*Bcajh97-01A*’ and ‘*Bcajh97-01B*’ and the high similarity of the coding sequences, we renamed *Bra011440* and *Bra037005* as *Brassica campestris Male Fertility 26a* (*BcMF26a*) and *BcMF26b*, respectively, in this study.

**Fig 3:**
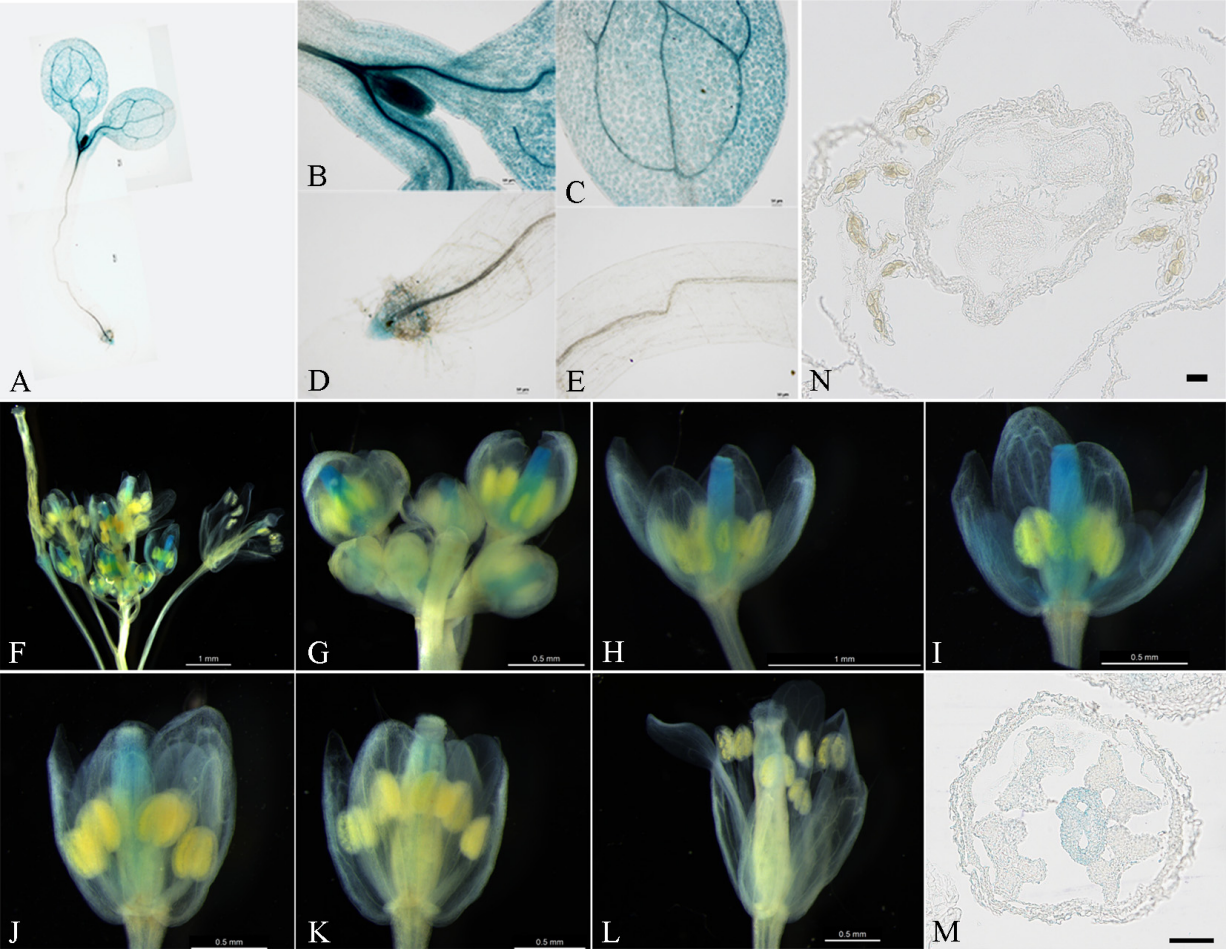
Spatial expression pattern of proBcMF26a: GUS-GFP. (A–L) Histochemical GUS assays of the T_2_ *Arabidopsis* transformed with pBGWFS7.0–proBcMF26a: GUS-GFP vector. (A–E) GUS assays in a four-day-old seedling. The GUS signals were detected in the (B) shoot apical meristem, (C) developing leaf, and (D) root tip. No GUS activity could be detected in (E) hypocotyl. (F–L) GUS assays in inflorescence of 35-day-old *Arabidopsis*. GUS activity was mainly detected in pistils. Moreover, (H–L) the GUS signal decreased with the growth of the pistils. (M–N) Transverse section of flower buds at the (M) pollen mother cell stage and (N) binucleate microspore stage. GUS activity mainly in pistils at the early stage of flower bud development was presented. Scale bars of O and P are 50 μm.

**Fig 4:**
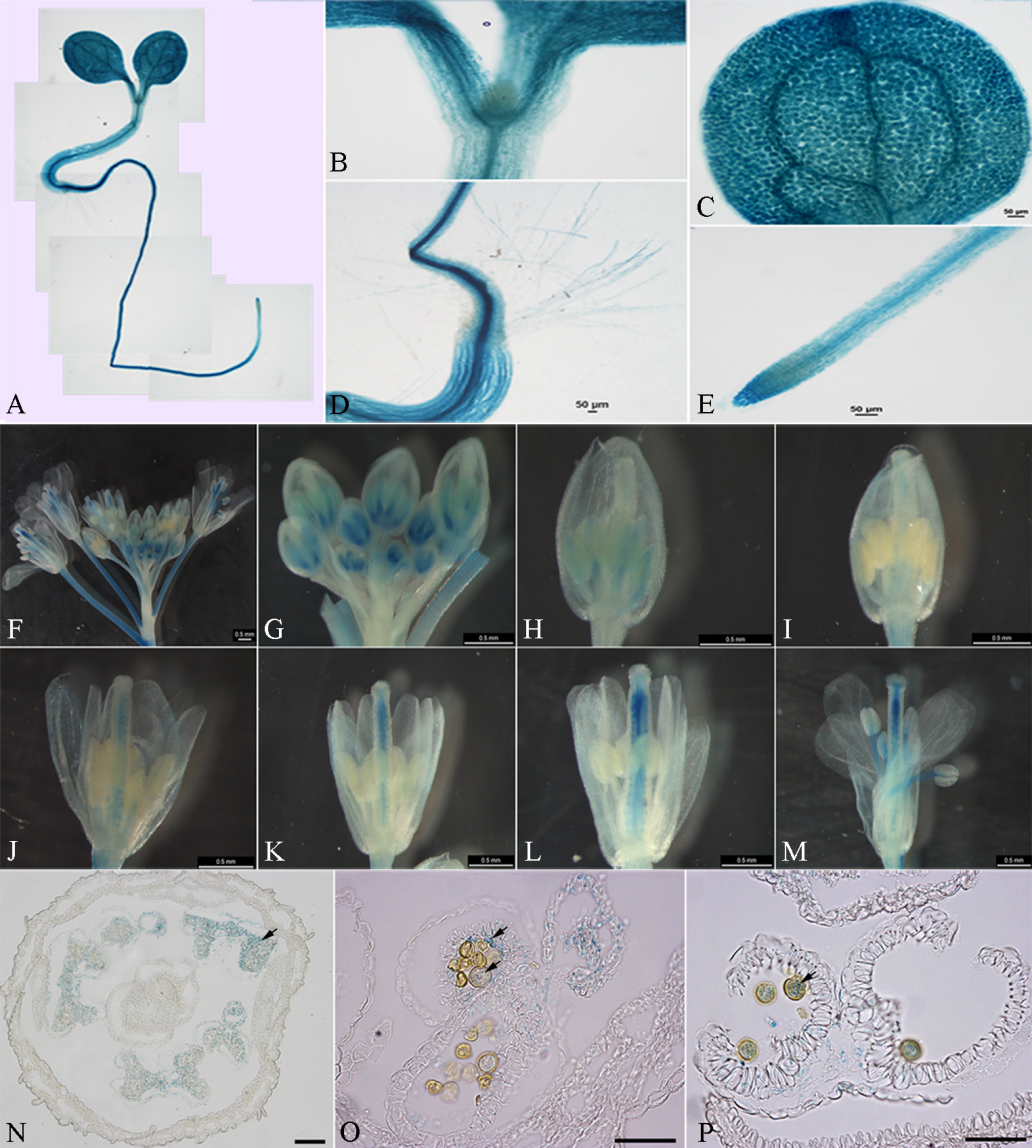
Spatial expression pattern of proBcMF26b: GUS-GFP. (A–M) Histochemical GUS assays of the T_2_ *Arabidopsis* transformed with pBGWFS7.0–proBcMF26b: GUS-GFP vector. (A–E) GUS assays in a four-day-old seedling. The expression signals were detected in all parts of the seedling, except for a weaker staining in (B) the shoot apical meristem. (F–M) GUS assays in inflorescence of 35-day-old *Arabidopsis*. GUS expression could be observed in (F) pedicels, in (G and H) anthers at the early stage of flower buds development, and in (K–M) pistils and filaments at the late stages of flower buds development. (N–P) Transverse section of flower buds at the (N) pollen mother cell stage, (O) binucleate microspore stage and (P) mature pollen stage, GUS activity was observed in anthers at the pollen mother cell stage, as well as in the tapetum and pollen grains at the binucleate microspore stage and mature pollen stage. Scale bars of N–P are 50 μm.

The fusion vectors pBGWFS7.0–proBcMF26a: GUS-GFP and pBGWFS7.0–proBcMF26b: GUS-GFP, were transiently transformed into onion epidermal cells to detect the activity of the promoter fragment. Typical GFP transient expression signals were observed in the onion epidermal cells (S5A and S5C Fig). By contrast, no signal was observed when the empty vector pBGWFS7.0: GFP was transformed. These results showed that the amplified promoter fragments demonstrated promoter activity. Meanwhile, GUS activity in the seedlings and inflorescences of the *Arabidopsis* T_2_ transgenic lines were examined (Figs 3 and 4). At the seedling stages, GUS activity driven by the *BcMF26a* promoter was observed in the shoot apical meristem, developing leaf, and root tip, no activity was observed in the hypocotyl (Fig 3A to 3E). The *BcMF26b* promoter drove GUS expression in all parts of the seedling (Fig 4A to 4E), although the signal in the shoot apical meristem was weak (Fig 4B). At the reproductive stages, obvious differences were observed in inflorescences. *BcMF26a* promoter drove GUS expression only in the pistils at the early stages of flower bud development, and the expression signal decreased with the growth of the pistils (Fig 3F to 3L). Whereas, *BcMF26b* promoter drove GUS expression in pedicels and anthers at the early stages of flower bud development, as well as in the pistils and filaments at the late stages of development (Fig 4F to 4M). The transverse sections of the flower buds at different flower bud developmental stages for *BcMF26a* (Fig 3M and 3N) and *BcMF26b* (Fig 4N to 4P) promoter-transformed plants also confirmed the results described above. *BcMF26a* promoter activity was still only observed the in pistils at the early developmental stages (Fig 3M). However, *BcMF26b* promoter activity was also detected in the tapetum at the binucleate microspore stage (Fig 4O) and in pollen grains at the mature pollen stage (Fig 4P). These results illustrate the partial overlapping at the seedling stages and spatial separation at the reproductive stages of *BcMF26a* and *BcMF26b* expression patterns. No GUS activity was observed in the empty vector transformed plants.

### BcMF26a and BcMF26b proteins could be localized to the cell wall

**Fig 5:**
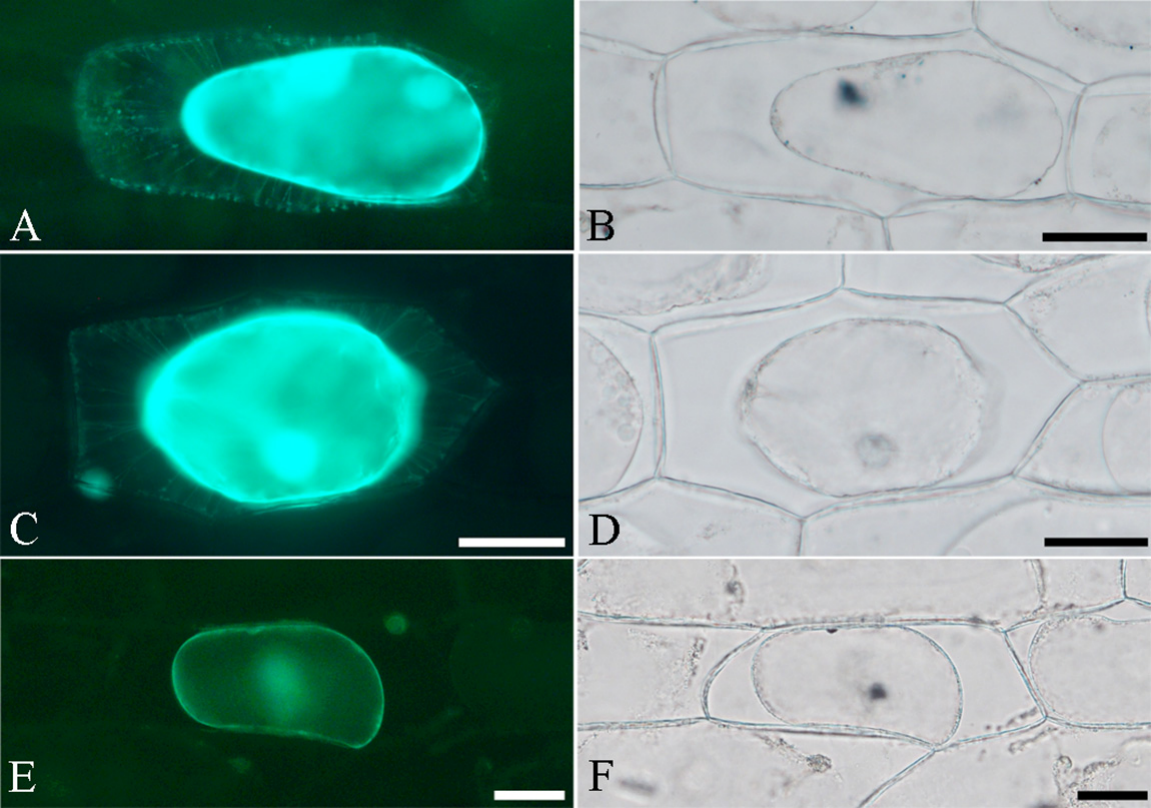
Subcellular localization of BcMF26a-GFP and BcMF26b-GFP fusion proteins in onion epidermal cells. (A, C, and E) Fluorescence images of plasmolyzed cells transformed with pFGC–BcMF26a: GFP, pFGC–BcMF26b: GFP, and pFGC: GFP, respectively. (B, D and F) Bright field image of the corresponding onion epidermal cells. (A and C) The fluorescence signal of the target proteins could be observed in the cytoplasm, cell wall, and space between the membrane and cell wall. (E) The fluorescence in the control cell could only be observed in the cytoplasm. Scale bars = 50 μm.

No signal peptide cleavage site was observed in the amino acid sequences of both BcMF26a and BcMF26b as predicted by SignalP 4.1 server. In the subcellular localization experiment, the cells transformed with pFGC–BcMF26a: GFP, pFGC–BcMF26b: GFP, and pFGC: GFP vectors showed obvious GFP signal in the whole cells. Then, the epidermal cells were plasmolyzed to determine whether the proteins can be localized to the cell wall. The GFP signal in the cells transformed with empty vector plasmid was only observed in the cytoplasm (Fig 5E). Whereas, the fluorescence signals in the cells transformed by the pFGC–BcMF26a: GFP and pFGC–BcMF26b: GFP vectors were observed in the cytoplasm, cell wall, and space between the cell membrane and cell wall (Fig 5A and 5C). Moreover, the fluorescence signals between the cell membrane and cell wall were distributed radially, linking the cell membrane and cell wall. These results indicated that BcMF26a and BcMF26b proteins can localize in cell wall.

### *BcMF26a* and *BcMF26b* co-inhibition led to abnormal pollen development and pollen tube growth

**Fig 6:**
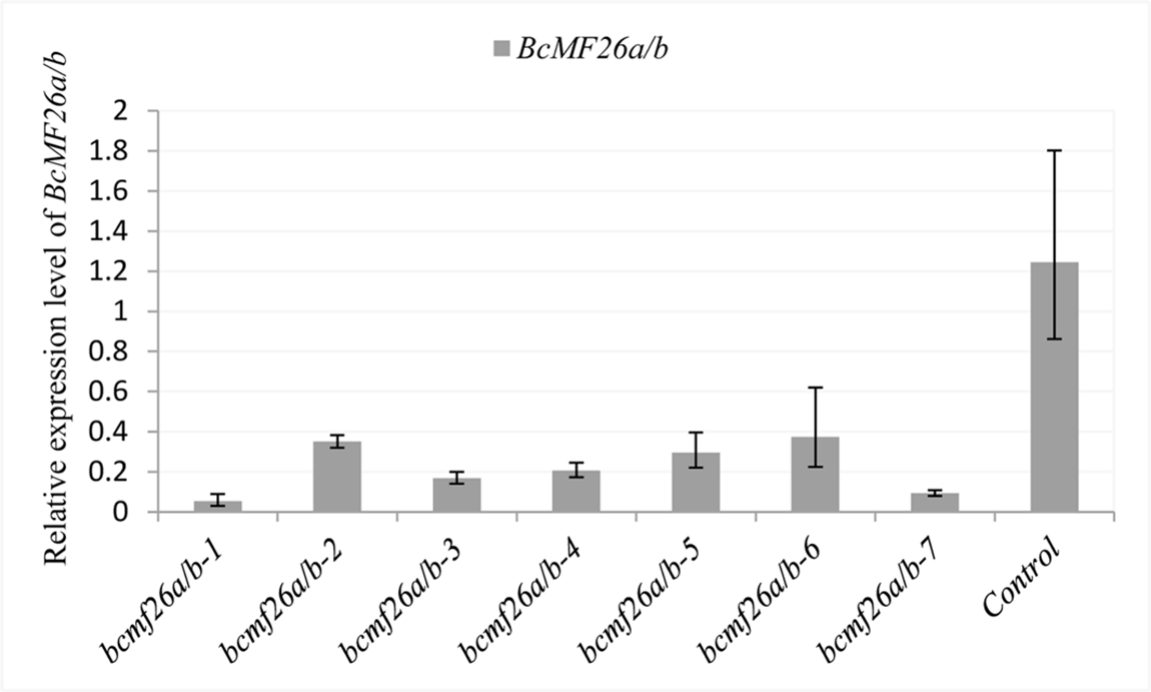
Analysis of the *BcMF26a/b* mRNA levels in the inflorescences of the *bcmf26a/b* and control lines using qRT-PCR analysis. The expression levels of *BcMF26a/b* in the inflorescences of *bcmf26a/b* lines were significantly less than that of the empty vector pCAMBIA1301-transformed plants (control). *UBC-10* was used as an internal control. Standard errors for three independent experiments are shown.

To investigate the biological role, multiple-target amiRNA construct co-inhibiting *BcMF26a* and *BcMF26b* was transformed into *B*. *campestris* ssp. *chinensis* var. *parachinensis* (S7A and S7B Fig, S8A to S8H Fig). Seven putative transgenic lines (*bcmf26a/b-1* to *bcmf26a/b-7*) were obtained after hygromycin screening. QRT-PCR analysis showed that the mRNA levels of *BcMF26a*/*b* in the inflorescences of the seven transformed lines were much lower than those in the control plants (Fig 6), indicating that the expression of the two genes was co-inhibited simultaneously and fairly.

**Fig 7:**
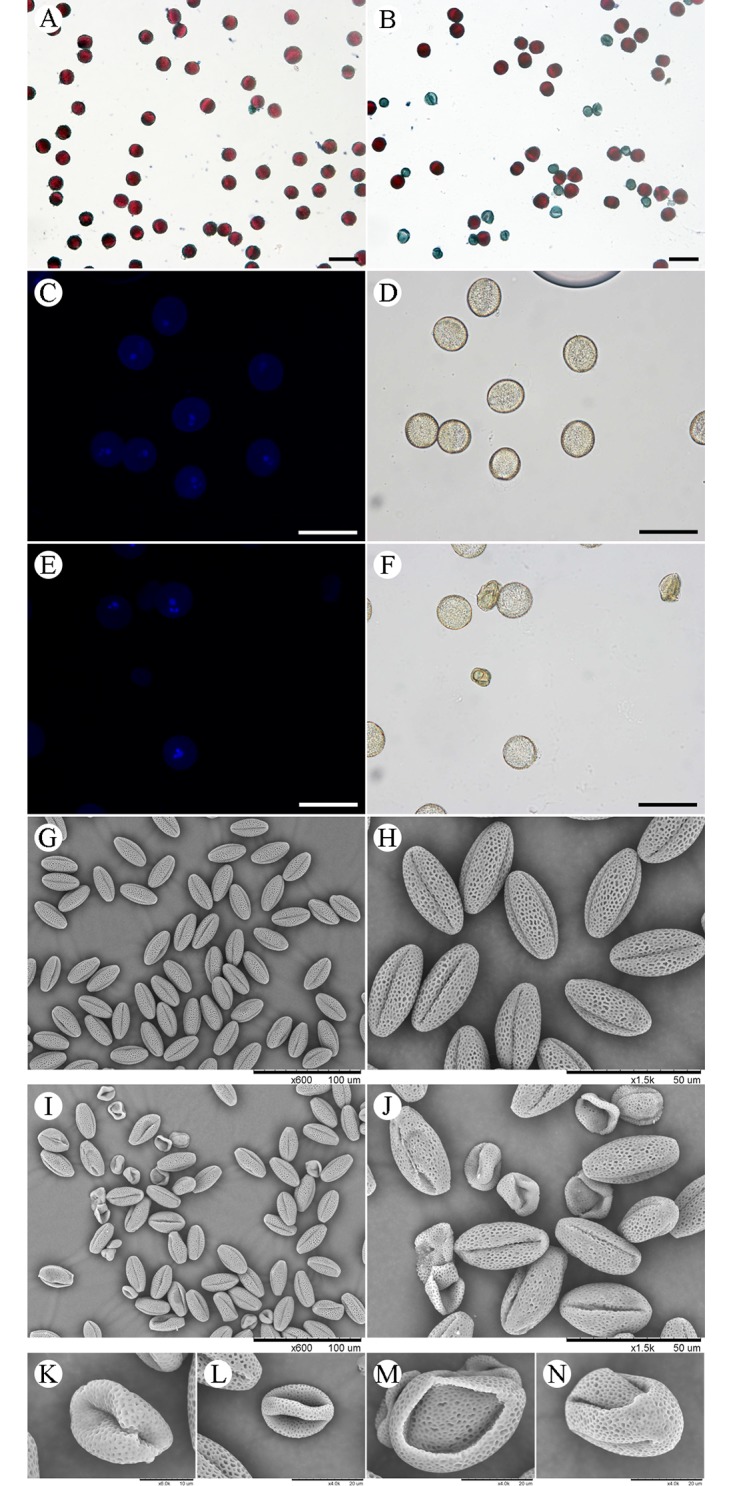
Pollen morphologies of the *bcmf26a/b* and control lines. (A and B) Alexander staining of pollen grains from the control plants and *bcmf26a/b* plants. (A) Mature pollen grains of the control plants were bright red upon staining with Alexander solution, whereas (B) the nonviable pollen grains from *bcmf26a/b* were blue-green. (C–F) DAPI staining observation of pollen grains from the (C and D) control plants and *bcmf26a/b* plants underfluorescence and (D and F) bright-field microscopy. (E and F) Mature pollen grains of control plants contained normal sperm nucleus and vegetative nuclei. Whereas, (C and E) the nuclei were absent in *bcmf26a/b* nonviable pollen grains. (G–N) SEM observation of pollen grains from control plants and *bcmf26a/b* plants. (G and H) (G and H) Mature pollen grains of control plants were uniformly spheroid and had finely reticulate ornamentation on their surface; whereas, (I–N) the irregular pollen grains from *bcmf26a/b* exhibited abnormal pollen wall and germinal furrows. Scale bars of A—F = 50 μm.

**Fig 8:**
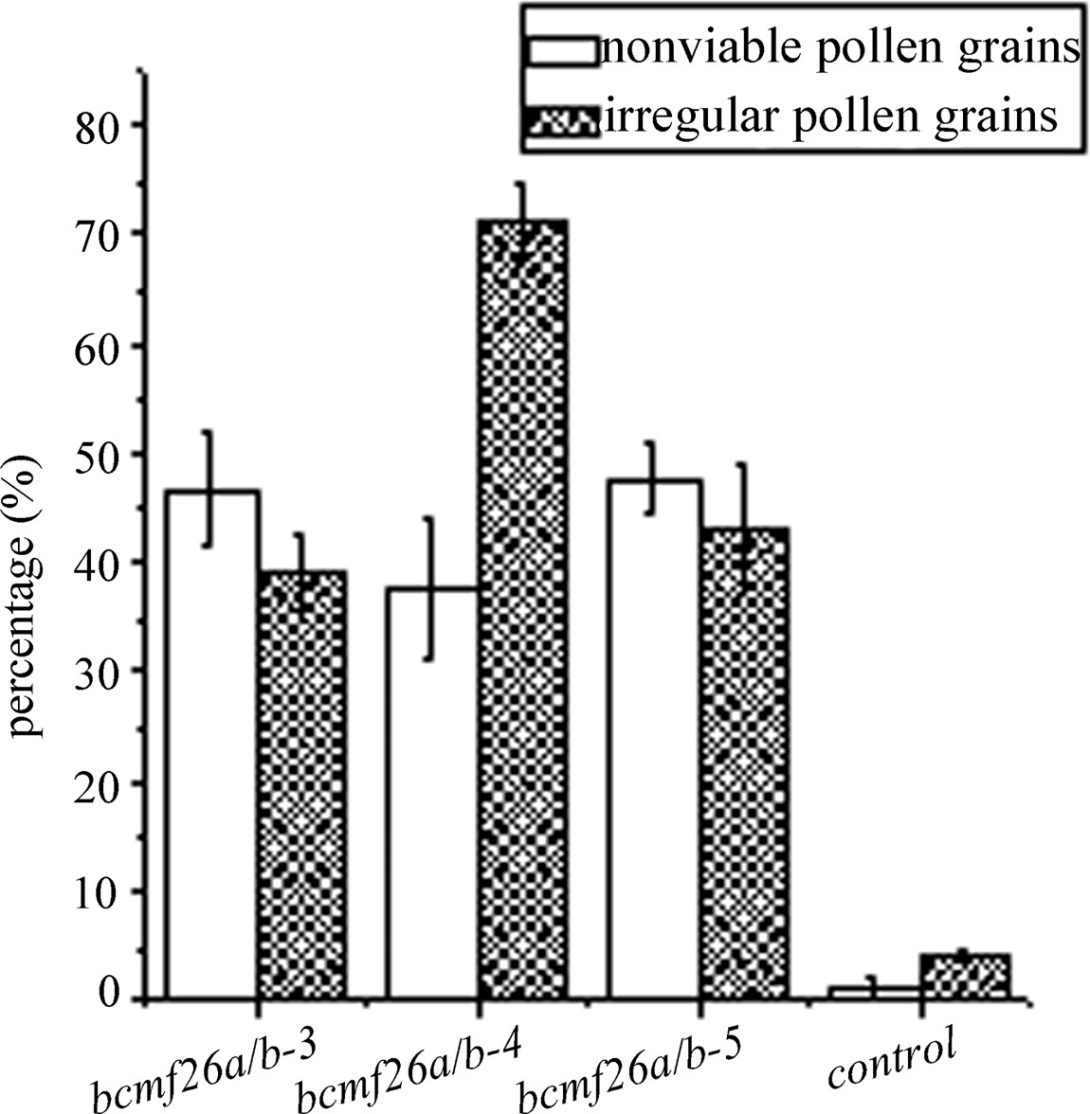
Viability percentage and irregular percentage of pollen grains in *bcmf26a/b* and control lines. Nearly half of pollen grains were nonviable in the *bcmf26a/b* plants, whereas the percentage was only 1.01% in the control. Approximately 38.9%–71.1% of the pollen grains were irregular in the *bcmf26a/b* plants, whereas ~3.75% was irregular in the control. Standard errors are shown.

The transgenic lines, *bcmf26a/b-3*, *bcmf26a/b-4*, *and bcmf26a/b-5*, which represent the average inhibitory level, were used for further analysis. The *bcmf26a/b* plants (S8G and S8I Fig) did not show defective phenotype at the vegetative development stage and the reproductive development stage compared with the control plants (S8H and S8J Fig). Meanwhile, the phenotypes of the floral organs in *bcmf26a/b* plants at the flowering stage were also normal (S8K, S8M, S8O, S8Q, and S8S Fig) compared with the control (S8L, S8N, S8P, S8R, and S8T Fig). Alexander staining was used to analyze pollen viability (Fig 7A and 7B). The statistical results showed that 37.5% to 47.8% of the *bcmf26a/b* pollen grains were nonviable, whereas this value was only ~2.1% for the control plants (Fig 8). Meanwhile, DAPI staining showed that the nonviable pollen grains of *bcmf26a/b* plants contained neither vegetative nuclei nor reproductive nuclei (Fig 7E and 7F). By contrast, the pollen grains from the control plants contained normal vegetative nuclei and reproductive nucleus (Fig 7C and 7D). SEM examination was used to detect the surface phenotype of pollen grains. The results revealed that mature pollen grains of the control plants were uniformly spheroid with finely reticulate ornamentation (Fig 7G and 7H). On the contrary, most of the pollen grains in the *bcmf26a/b* lines formed irregular clumps and exhibited abnormal reticulate ornamentation and germinal furrows (Fig 7I to 7N). Statistically, 38.9% to 71.1% of *bcmf26a/b* pollen grains exhibited irregular shapes, but this percentage was only ~3.75% for the control plants (Fig 8).

**Fig 9:**
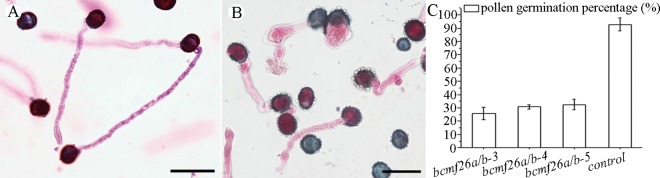
Germination analysis of pollen grains *in vitro* in control and *bcmf26a/b* lines. (A and B) Morphological characterization of pollen germination. (A) Pollen tubes from control plants germinate normally. (B) The nonviable pollen grains of the *bcmf26a/b* lines could not germinate; the germinated pollen tubes of the *bcmf26a/b* lines exhibited two defective morphologies: pollen wall burst at the beginning of germination and pollen tube tip burst during the elongation process. Images of pollen tubes were obtained at 4 h after germination. Scale bars = 50 μm. (C) Comparison of pollen germination percentage between the pollen of *bcmf26a/b* and control plants. The percentage of normal pollen tube was only 27.7%–35.1% in the *bcmf26a/b* lines, but it was ~92.8% in the control. Standard errors are shown.

*In vitro* pollen germination analysis showed none of the germinated pollen tubes of the control pollen grains burst or displayed abnormal shapes after 4 h of incubation (Fig 9A). The germination rate was ~92.8% (Fig 9C). Whereas, in the *bcmf26a/b* lines (Fig 9B), the nonviable pollen grains could not germinate; the germinated abnormal pollen tubes exhibited two defective morphologies: pollen wall burst at the beginning of germination and pollen tube tip burst during the elongation process. The percentage of normal pollen tube was only 27.7% to 35.1% for the *bcmf26a/b* lines (Fig 9C).

### *BcMF26a* and *BcMF26b* co-inhibition led to pollen deformities with abnormal intine development

**Fig 10:**
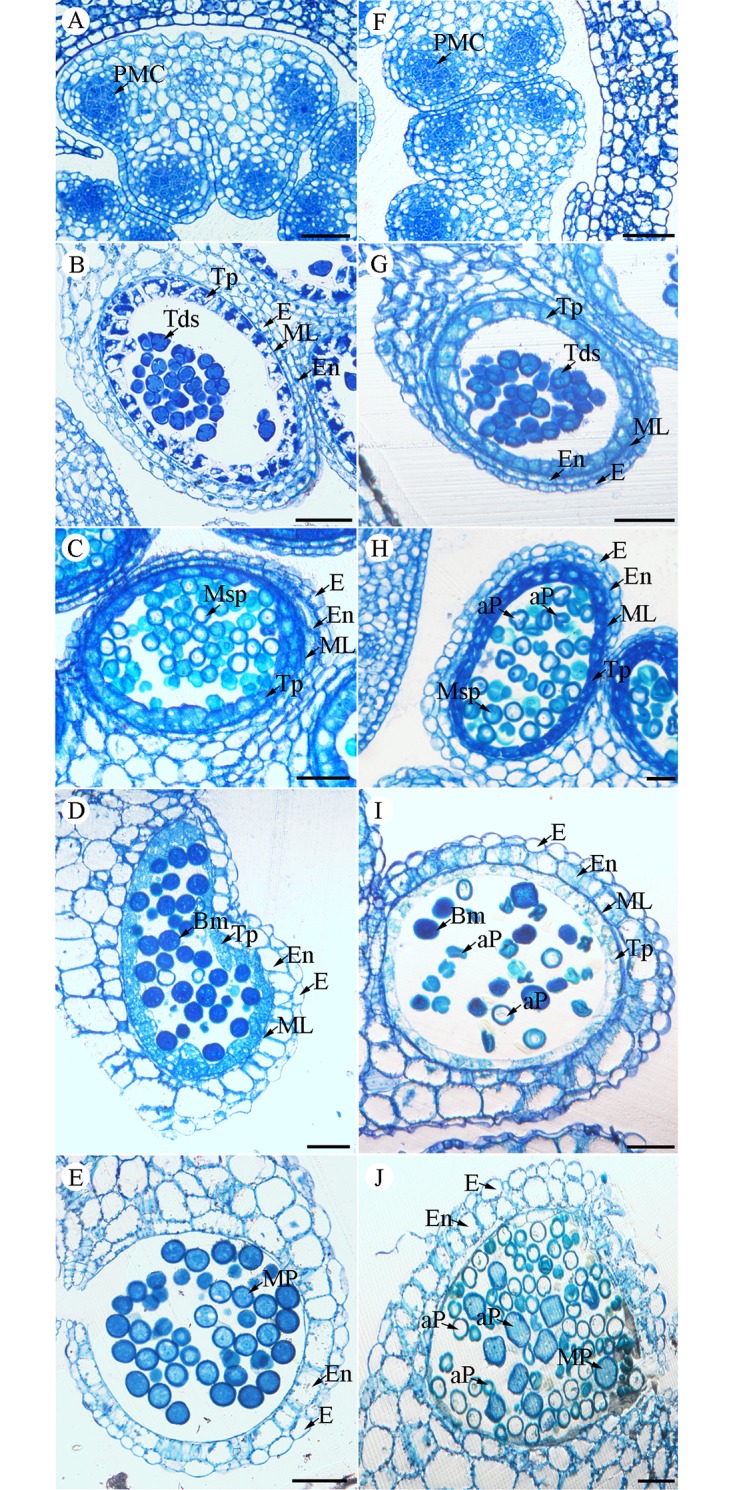
Comparison of transverse sections of pollen development in the control and *bcmf26a/b* anthers. (A to E) Semi-thin sections of anthers from the control plants. (F to J) Semi-thin sections of anthers from *bcmf26a/b* transformed plants. The pollen at the (A and F) pollen mother cell stage, (B and G) tetrad stage, (C and H) uninucleate stage, (D and I) binucleate microscope stage, (E and J) trinucleate microscope stage were observed. No obvious difference in pollen mother cell stage and tetrad stage was observed. The differences in pollen morphology between the control and *bcmf26a/b* were observed from the uninucleate microscope stage to the trinucleate microscope stage. aP, aborted pollen; E, epidermis; En, endothecium; ML, middle layer; Msp, microspore; P, pollen; Tp, tapetum; Tds, tetrads; Bm, binucleate microscope; MP, mature pollen. Scale bars = 50 μm.

Semi-thin section analysis of the anthers for the control and *bcmf26a/b* lines was performed to further clarify pollen development of the transgenic plants (Fig 10). At Stage I and Stage II, no obvious differences were observed in the anthers between the control plants (Fig 10A and 10B) and the *bcmf26a/b* lines (Fig 10F and 10G). At Stage III, uninucleate microspores with large vacuoles were distributed in the anther locules of the control anthers (Fig 10C), whereas most of the microspores shrunk and deformed in the *bcmf26a/b* anthers (Fig 10H). At Stage IV, the control anthers were filled with well-developed pollen grains, and the tapetum began to degenerate (Fig 10D). However, the *bcmf26a/b* pollen grains exhibited abnormal shapes, indicating shriveled, vacuolated, and swollen phenotypes (Fig 10I). The anthers became dehiscent and mature pollen grains were densely stained at Stage V in the control plants (Fig 10E). The surviving defective pollen grains in the *bcmf26a/b* lines were shriveled, swollen with dense staining, or vacuolated and could not be stained (Fig 10J).

**Fig 11:**
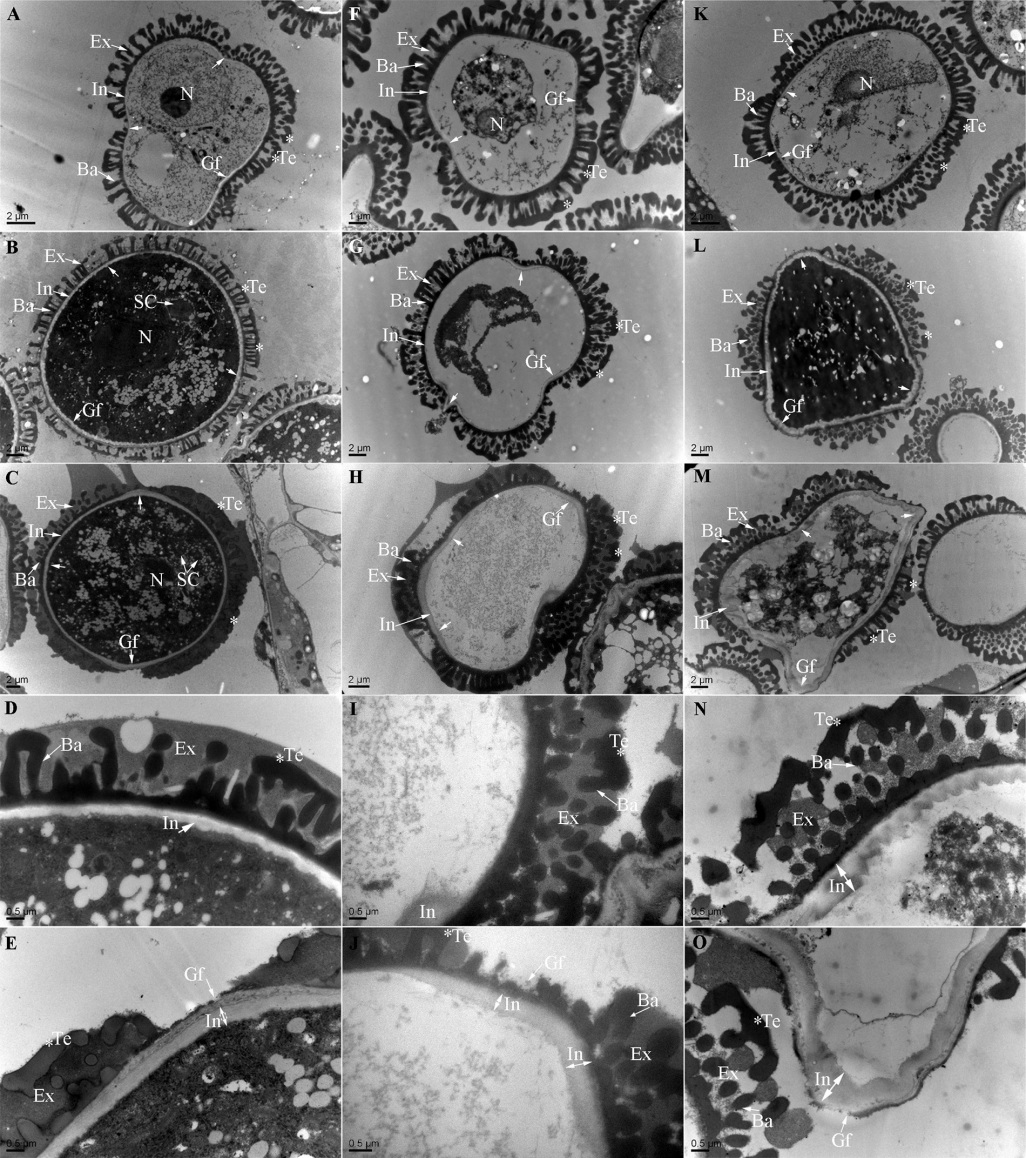
Transmission electron micrographs of microspores from the control plants and *bcmf26a/b* lines. Pollen at the (A and F) uninucleate microscope stage, (B and G) binucleate stage, and (C and H) mature pollen stage of the control plants and *bcmf26a/b* plants were observed. (A–C) The control pollen grains showed normal morphology with three germinal furrows and a normal development process of the pollen wall; the pollen intine (D) outside and (E) inside the germinal furrow region developed normally. (F–O) The pollen grains from the *bcmf26a/b* lines showed two kinds of deficient phenotype. One ultimately produced (F–H) vacuolated pollen grains with disordered formation of the intine. The pollen intine (I) disappeared in some regions, while the intine was (J) abnormally thick in other regions. The other kind of deficient phenotype was the formation of (K–M) swollen pollen grains. The development of pollen cytoplasmic inclusions was abnormal. Remarkable thickening (N) outside and (O) inside the germinal furrow regions was observed, compared with the control pollen. Ba, baculum; Ex, exine; Gf, germinal furrow; In, intine; N, nucleus; SC, sperm cell; Te, tectum.

Anther transverse sections were observed by TEM. The differences between the control and *bcmf26a/b* pollen were observed at uninucleate, binucleate, and trinucleate stages during intine formation. For the control pollen at uninucleate stage (Fig 11A), the exine layers took shape and underwent further thickening, whereas no exine deposition occurred in the regions of the three prospective germinal furrows; meanwhile, the outer and inner layers of intine began to form, which were much thicker in the germinal furrows than that in the other regions. At the binucleate stage (Fig 11B), intine within the germinal furrows underwent further thickening. At the trinucleate mature pollen stage (Fig 11C), the shape of intine both outside (Fig 11D) and inside (Fig 11E) the germinal furrow regions was normal; the vegetative nuclei and generative nucleus also formed normally. Compared with the control pollen, the *bcmf26a/b* pollen mainly exhibited two kinds of deficient phenotype: vacuolated pollen grains (Fig 11F to 11H) and swollen pollen grains (Fig 11K to 11M), with percentages of ~43.7% and ~10%, respectively. For the formation of the vacuolated pollen grains, the pollen cytoplasmic inclusions began to degrade at the uninucleate stage (Fig 11F), and the nuclear membrane was shriveled. Continuously, the nuclei was disappeared at binucleate stage (Fig 11G), although some cytoplasmic residues still could be observed. At the mature pollen stage (Fig 11H), the pollen cytoplasmic inclusions completely disappeared. Moreover, the development of intine was disorderly. Intine disappearance (Fig 11I) or abnormal thickening (Fig 11J) out of the germinal furrows was detected. For the formation of the swollen pollen grains, the development of pollen cytoplasmic inclusions and the differentiation of the vegetative nuclei and generative nucleus were abnormal. At the binucleate stage (Fig 11L), the pollen grain was filled with dense cytoplasm, no obvious vegetative nuclei and generative nucleus was observed. Moreover, the germinal furrows were pointing outward. At the trinucleate stage (Fig 11M), pollen cytoplasmic inclusions further changed, with number of spherical substances. Meanwhile, the intine outside (Fig 11N) and inside (Fig 11O) the germinal furrow regions exhibited abnormal thickening compared with the control pollen. No obvious difference in the *bcmf26a/b* anther was observed at Stage I and Stage II compared with that in the control: the nuclei exhibited normal division and differentiation, and the cell organelles formed normally.

### *At4g33440* was required for normal pollen tube growth

**Fig 12:**
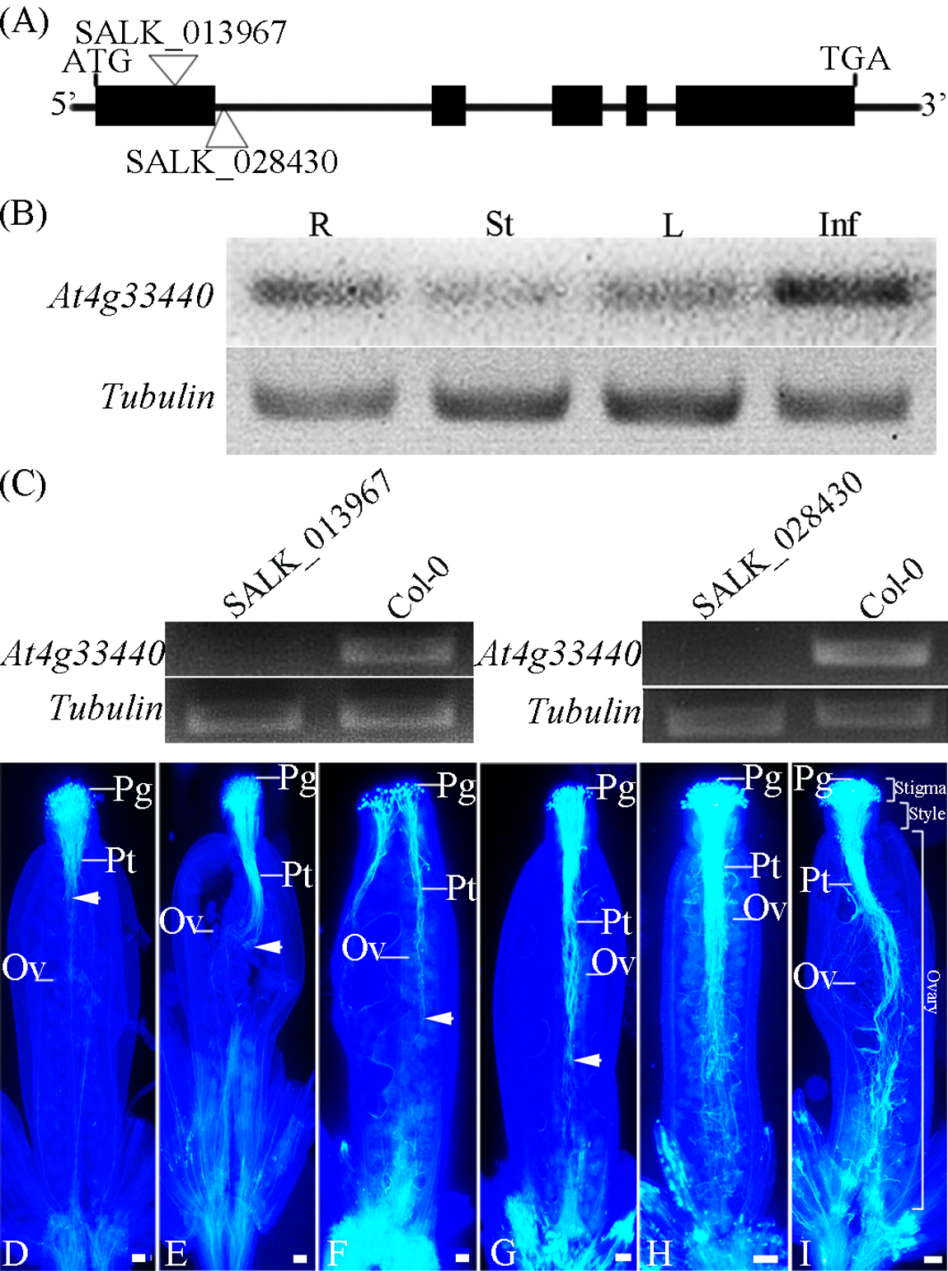
Gene structure and expression characteristic analysis of *At4g33440* and the T-DNA insertion mutants. (A) The gene structure of *At4g33440* and T-DNA insertion sites of two allelic mutants. The T-DNAs were inserted into the second intron and the first exon of *At4g33440* to produce SALK_028430 and SALK_013967, respectively. (B) *At4g33440* transcript level was detected in roots (R), stems (St), leaves (L), and inflorescences (Inf) by RT-PCR in 35-day-old plants in wild-type plants (Col-0). (C) The mRNA of *At4g33440* was not detected in SALK_028430 and SALK_013967. *Tubulin* was used as an internal control. (D–G) *In vivo* pollen tube growth in the pistils of the SALK_013967 lines. (D, F, and H) Pollen grains of SALK_013967 lines were pollinated onto the stigmas of the SALK_013967 lines. (E, G and I) Pollen grains from the wild type-plants were pollinated onto the stigmas of the SALK_013967 lines. (D and E) Growth status of pollen tubes after 4 h of incubation. (F and G) Growth status of pollen tubes after 12 h of incubation. (H and I) Growth status of pollen tubes after 24 h of incubation. The white arrows indicate the positions of the longest pollen-tube tips for each pistil in the transmitting tract. At 4 and 12 h after pollination, (D and F) the SALK_013967 pollen tubes grew obviously slower than (E and G) the pollen tubes from the wild-type plants. At 24 h after pollination, both (F) the SALK_013967 pollen tubes and (G) the wild type pollen tubes reached the bottom of the pistils. Pt, pollen tube; Ov, ovule; Pg, pollen grain. Scale bars = 100 μm.

*At4g33440* is the orthologous gene of *BcMF26a* and *BcMF26b*. In the wild-type plants of *A*. *thaliana*, *At4g33440* had higher expression levels in inflorescences compared with those in roots, stems, and rosette leaves, as confirmed by RT-PCR analysis (Fig 12B). The two knockout mutants of *At4g33440* (SALK_028430 and SALK_013967) were characterized (Fig 12A and 12C) to better investigate the biological functions of *BcMF26a* and *BcMF26b* and take advantage of the T-DNA pool. The growth status of plants, Alexander staining of pollen grains, and seed numbers in the mature siliques showed no obvious differences between the SALK_013967 mutant and wild-type plants (S6 Fig).

*In vivo* pollen germination experiment was performed to characterize pollen tube movement. The pollen grains from wild-type plants and SALK_013967 mutant were hand-pollinated on the pistils of the SALK_013967 mutant. After aniline blue staining, the pollen tubes in the pistils were visualized by fluorescent microscopy. After 4 h of pollination, pollen tubes that germinated from wild-type pollen grains traveled a substantial distance into the transmitting tract in the SALK_013967 pistils (Fig 12D), whereas, those that germinated from the SALK_013967 mutant pollen grains only slightly penetrated the ovary chamber (Fig 12E). After 12 h of incubation, the pollen tubes that germinated from the SALK_013967 mutant pollen grains penetrated approximately half of the way into the ovary chamber (Fig 12F), whereas those that germinated from the wild-type pollen grains were penetrated longer (Fig 12G). However, the pollen tubes that germinated from the wild-type and SALK_013967 mutant pollen grains could reach the bottom of the transmitting tracts at 24 h after pollination (Fig 12H and 12I). These findings indicated that the absence of *At4g33440* may delay pollen-tube growth to some extent, but not affect the final fertilization and seed numbers. Similar results were also observed in the SALK_028430 mutant.

## Discussion

### *BcMF26a* and *BcMF26b* are PG genes that originated from a segmental chromosomal duplication

PG genes contain four motifs (motif I, SPNTD; motif II, GDDC; motif III, GPGGHG; motif IV, RIK) and belong to the glycosyl hydrolase family 28 [39]. They were classified as endo-PGs, exo-PGs, and rhamno-PGs. A total of 225 PG genes were grouped as endo-PGs in Clades A and B, exo-PGs in Clades C and D, and rhamno-PGs in Clade E, the Clade F members could not be clearly defined as either endo- or exo-PGs [22]. PG genes belonging to Clade E are ubiquitously expressed and only contain three typical structure motifs (I, II, and IV) [22, 23]. *BcMF26a* and *BcMF26b* are orthologous genes of the PG gene *At4g33440* that contain motifs I, II, and IV of PG protein. Moreover, phylogenetic analysis indicated that *BcMF26a* and *BcMF26b* are clustered into Clade E with other PG genes from different species. Thus, *BcMF26a* and *BcMF26b* are two putative PG genes and may be with rhamno-PGs activity. These characteristics are similar to the PG gene *BcMF24* previously cloned in *B*. *campestris* [40].

The mechanisms of gene duplications are as follows: local (tandem) duplication, tetraploidy (or polyploidy), chromosomal segmental duplication, and single gene transposition-duplication [41, 42]. Among these mechanisms, chromosomal segmental duplication covers 89% of the *Arabidopsis* genome [43]. It is defined as a number of genes of a whole chromosome are duplicated as a segment and they begins with the fractionation process [42]. The retained, segmentally duplicated genes are called syntenic paralogs. According to the results, the regions closely surrounding *BcMF26a* and *BcMF26b* on chr1 and chr3 are syntenic conserved chromosomal blocks. These regions are interspersed and rearranged as fragments with high similarity, suggesting that *BcMF26a* and *BcMF26b* originated as a result of a chromosomal segmental duplication.

### Differential expression characteristics of *BcMF26a* and *BcMF26b* are probably caused by the differences in promoter and intron sequences

The *BcMF26a* and *BcMF26b* promoters can drive the GUS signal in leaves and root tips of seedlings. In addition, their orthologous gene *At4g33440* can express in roots, stems, leaves, and inflorescences. Thus, both *BcMF26a* and *BcMF26b* retained some expression characteristics of *At4g33440* during the evolutionary process. The expression level of *BcMF26b* was much higher than that of *BcMF26a* in all the organs and tissues. Moreover, *BcMF26a* was hardly expressed in all the detected organs and tissues. Meanwhile, at the seedling stages, the *BcMF26b* promoter could drive GUS expression in hypocotyl, whereas the *BcMF26a* promoter could not. At the reproductive stages, GUS activity of *BcMF26b* was observed in the pedicels, pollen grains, tapetum, pistils, and filaments, whereas the *BcMF26a* promoter only drove GUS signal in the pistils. Therefore, *BcMF26a* and *BcMF26b* are two duplicated genes with expression divergences.

Duplicated genes initially have identical sequences and functions but tend to diverge in regulatory and coding regions. Divergence in regulatory regions can result in shifts in expression pattern [44]. Genes with the same biochemical functions may also be expressed at different times or in different places [45]. *ATX1* and *ATX2* were two highly conserved duplicated genes in *Arabidopsis*. Although structurally similar, their regulatory sequences differed, resulting in distinct temporal and spatial expression patterns [46]. *BcMF26a* and *BcMF26b* had the same number of exons and introns. They shared significant homology in their ORFs and amino acid sequences. However, the regulatory sequences upstream of ‘ATG’ were significantly different. Furthermore, changes in *cis*-regulatory modules (promoters) of duplicated genes may lead to specific shifts in expression patterns between duplicated genes [47, 48]. Therefore, the differential expression patterns between *BcMF26a* and *BcMF26b* are probably caused by the differences between their prompter regions. Moreover, duplicated genes show significant homology within coding regions, but none within introns, may also have differences in expression patterns and functions. For example, two maize duplicated *FIE* genes (*FIE1* and *FIE2*) share significant homology over their coding regions, but their expression pattern significantly differ because of the differences in their *cis*-acting elements and introns [49]. Meanwhile, Xu et al. found that divergences in exon–intron structure have been very prevalent in duplicated genes and, in many cases, have led to the generation of functionally distinct paralogs [44]. *BcMF26a* and *BcMF26b* have the same numbers of exons and introns, and the intron phases are also consistent. However, the lengths and sequence similarities of the four introns between the two genes were largely divergent. Therefore, the divergences of introns may also contribute to the generation of different expression patterns between *BcMF26a* and *BcMF26b*. For these hypothesizes, more experiments will be needed.

### BcMF26a and BcMF26b proteins may play roles for cell wall construction and secret by unconventional secretory mechanism

PG proteins involving in the degradation of pectin are considered to be localized in the cell wall [50]. Compared with the control, subcellular localization of BcMF26a and BcMF26b proteins showed that the fluorescence signals could be localized to the cell wall. Therefore, BcMF26a and BcMF26b may play roles in cell wall construction. The processes of eukaryotic protein secretion include two mechanisms: signal peptide-dependent secretory transport and unconventional protein export [51]. Although a number of secretory proteins with defined extracellular functions do not possess functional signal peptides, they are transported to extracellular space by unconventional protein export [52]. In the present study, no signal peptide cleavage site was present in amino acid sequences of BcMF26a and BcMF26b proteins by SignalP 4.1 server prediction. Thus, they may be released by unconventional protein export. Furthermore, in the plasmolyzed onion epidermal cells, the fluorescence signal was also observed in the cytoplasm. It can be inferred that the proteins were transported by vesicles which fused with the plasma membrane. The fluorescence signal existed in the cell space between the cell membrane and cell wall. Moreover, it was radially distributed, linking the cell membrane and cell wall. This may be the method by which BcMF26a and BcMF26b proteins move from the plasma membrane to the cell wall.

### *BcMF26a/b* impairs pollen development and pollen tube growth by modulating intine information

The analysis of complete eukaryotic genome sequences has revealed that gene duplication is indeed thriving [53]. Furthermore, many eukaryotic organisms have had their whole genome duplicated, sometimes more than once, such as *Brassiceae* [54]. Functional redundancy or complementarity has occurred among many genes. Natural plant miRNAs have a very narrow action spectrum and target only mRNAs with few mismatches [34]. With highly specific gene silencing by amiRNAs, single-, double-, and triple-mutant analysis have been used for redundancy studies [55]. Such studies inspired us to inhibit *BcMF26a* and *BcMF26b* independently and specifically using amiRNA technology so that their functions can be studied. Unfortunately, *BcMF26a* and *BcMF26b* shared high similarity over their ORFs, and the length of continuous differential bases in the ORFs could not meet the designed requirements for single-inhibition. Considering that PG was encoded by a large gene family in plants, we designed the multiple-target amiRNA to inhibit the expression of *BcMF26a* and *BcMF26b* simultaneously. This design excluded the functional redundancy of other PG genes in the family.

The PG genes involving in pollen wall development are considered to be necessary for pollen development and pollen tube formation [56]. In the T-DNA insertion mutants of *At4g33440*, pollen tubes growth was delayed. A similar phenomenon was observed in the mutants of *COBRA-LIKE 10* (*COBL10*), which caused gametophytic male sterility because of reduced pollen tube growth and compromised directional sensing in the female transmitting tract [57]. Obvious defective phenotypes were also observed in *bcmf26a/b*, with pollen viability of only 37.5% to 47.8% and the germination rate of normal pollen tube was only 27.7% to 35.1%. Therefore, the absence of *BcMF26a/b* seriously impaired pollen development and pollen tube growth.

Pectin is a major component of pollen intine, and it is also enriched at the germinal furrows [3, 58]. With LM5 mAb, pectin was immunolocalized in pollen and pollen tube of olive, especially at all the three apertures [59]. As a hydrolase and loosening enzyme, PG disintegrates the cell wall structure through pectin degradation [6]. According to anther semi-thin section analysis, the most dramatic aberrations in the *bcmf26a/b* transgenic plants were pollen deformities characterized by swelling with dense staining and vacuoles that could not be stained. TEM revealed that the pollen grains underwent a disordered formation in the intine region. Abnormal thickening of the intine inside and outside the germinal furrows was observed. Meanwhile, the development of cytoplasmic materials and nucleus was also disorderly. Given that intine synthesis is largely under the control of the microspore [60], it is credible that the cytoplasmic inclusions and intine are disordered simultaneously. Therefore, *BcMF26a/b* may affect pollen development by disrupting intine formation. These aforementioned phenotypes are also similar to the *osgt1* and *cap1* mutants, which affected pollen development and maturation by modulating the abnormal intine structure. *GLYCOSYLTRANSFERASE1* (*OsGT1*) encodes glycosyltransferase, which is essential for intine construction and pollen maturation; in *osgt1* pollen, the intine structure was disrupted and the grains were shrunken [61]. The *collapsed abnormal pollen1* (*cap1*) mutant produces abnormal pollen grains, which lack almost all cytoplasmic materials, nuclei, and intine, and cannot germinate [62].

During pollen germination, the outer layer of the pollen tube wall appears to be an extension of the pectinaceous intine of the pollen grain [63]. The apical wall layer is rich in methylated pectic to allow cell expansion, and the shank wall consists of demethylated pectin [64]. Shortly after pollen grains are exposed to water, the exine abruptly breaks with pressure accumulated from the expansion of the intine; in optimal conditions, the germinating pollen cell emerges from one end of the pollen grain and then travels through the appropriate germinal furrow of the intine [13]. During *in vitro* germination, the *bcmf26a/b* pollen tubes burst at the beginning of the germination, or the pollen tube’s tip burst during the elongation process. Those abnormal morphologies are possibly caused by the thickened intine, which may increase the pressure for pollen germination and pollen tube growth.

*BcMF2* and *BcMF9* are two PG genes in *B*. *campestris*. Based on our obtained phylogenetic tree, these two genes both belonged to Clade C. *BcMF2* was specifically expressed in the tapetum and pollen after the tetrad stage; in *bcmf2* plants, mature pollen presented a distorted morphology in the intine, leading to abnormal pollen tube growth and a consequent reduction in male fertility [13]. *BcMF9* was also expressed in the tapetum and microspores during the late stage; it affected pollen and pollen tube development by playing a role in intine and exine formation [14]. In the present study, *BcMF26a* and *BcMF26b* are also PG genes in *B*. *campestris*, but they belong to Clade E. In *bcmf26a/b* plants, mature pollen also presented a distorted morphology in the intine. However, the expression of *BcMF26b* in anthers was at the early stages of pollen development. Moreover, *BcMF26b* did not specifically express in anther; for example, the activity of its promoter was also observed in pedicels. Whether the expression of the target genes in other organs also contributes to the phenotypes of *bcmf26a/b* plants has not been verified.

### *BcMF26b* may play a major role in pollen development of the *bcmf26a/b* plants

In the T-DNA insertion mutants, the absence of *At4g33440* could only delay the growth rate of pollen tubes, whereas, both the pollen grains and pollen tubes all showed defective phenotypes in *bcmf26a/b* transgenic plants. Therefore, *BcMF26a/b* may be the result of neofunctionalization during evolution. According to our results, the expression level of *BcMF26b* was much higher than that of *BcMF26a* in all the tested organs and tissues. The *BcMF26a* promoter activity was only observed in pistils at the early stages of flower buds development, while *BcMF26b* promoter could drive GUS signal in anther and pollen. Therefore, *BcMF26b* may play major roles in pollen development and pollen tube formation in *bcmf26a/b* plants, though we designed multiple-targeted amiRNA to realize co-inhibition. In addition, *bcmf26a/b* plants had normal pistils consistent with the control plants during the flowering period. Thus, *BcMF26a* gene may have a weaker function that could not affect the growth and development of plants. Furthermore, cell separation has been recognized for its involvement in plant development, which includes pod abscission and dehiscence [65]. Although the *BcMF26b* promoter could drive GUS expression in pistils and filaments at the late stages of flower buds development, the morphologies of pistils and filaments in *bcmf26a/b* plants were normal. This finding may be due to the possible that *BcMF26b* also participates in the senescence and abscission of pistils and filaments as a cell wall hydrolytic enzyme, so it cannot affect the early growth and development of the two organs.

## Supporting Information

S1 Fig Gene structure, nucleotide sequence, and amino acid sequence analysis. (A) The positions of exons and introns are shown. Both of them contain five exons (black boxes) and four introns (thin lines). The numbers above indicate the intron phases. (B) Nucleotide sequence alignment of *BcMF26a* and *BcMF26b*. The sequences of the two genes share high similarity. The positions of the primers used for qRT-PCR are shown for *BcMF26a* and *BcMF26b* with red boxes and yellow boxes, respectively. The locations of the start codon are marked by green boxes. (C) Amino acid sequence alignment of *BcMF26a* and *BcMF26b*. The amino acid sequences of the two genes have high similarity. The locations of the four conserved motifs of PG protein are marked by red boxes. *BcMF26a*, *BcMF26b*, and *At4g33440* all contained three out of the four typical conserved domains.
(TIF)

S2 Fig Multiple sequence alignment analysis of the deduced intron sequences. (A–D) Alignment results of the intron sequences between *BcMF26a* and *BcMF26b* by ClustalX software. The lengths and sequence similarities of the four pairs of introns have large divergences.
(TIF)

S3 Fig Multiple sequence alignment analysis of the deduced regulatory sequences upstream of ‘ATG’. The significant divergences are presented. *BcMF26a-U* and *BcMF26b-U* indicate the names of deduced regulatory sequences upstream of ‘ATG’.
(TIF)

S4 Fig Multiple sequence alignment analysis of the deduced amino acid sequences. *BcMF26a*, *BcMF26b*, and *At4g33440* contain three out of the four typical conserved domains of PG protein.
(TIF)

S5 Fig Promoter transient expression analysis in onion epidermal cells. (A and C) GFP signal in the onion epidermal cells transformed with pBGWFS7.0–proBcMF26a: GUS-GFP and pBGWFS7.0–proBcMF26b: GUS-GFP vectors, respectively. (B and D) Bright field images of the corresponding onion epidermal cells. Scale bars = 50 μm.
(TIF)

S6 Fig Phenotype observation of the wild-type *Arabidopsis* plants and SALK_013697 mutant. (A) Growth status of the wild–type and SALK_013697 plants at the rosette leaf stage, respectively. (B) Growth status of 35-day-old plants of the wild–type plants and SALK_013697 mutants, respectively. (C and D) Growth status of the flowers of the wild–type plants and SALK_013697 mutants, respectively. (E and F) Alexander staining of pollen grains of the wild type plants and SALK_013697 mutants, respectively. No obvious difference was observed between the mutant and the wild type.
(TIF)

S7 Fig The sequence and folding structure of amiRNA. (A) Sequence used for constructing the amiRNA. The amiRNA was constructed based on the structure of MIR164a. The bases marked with green shadow indicate the selected sequence for constructing the amiRNA of *BcMF26a/b*; the bases marked with red shadow indicate its reverse complemented sequence. The blue and red bases indicate the restriction enzyme sites XbaI and HindIII, respectively. (B) The RNA folding form of the amiRNA, which was predicted by the mfold Web Server.
(TIF)

S8 Fig Transgenic plant obtained and morphological observation of control and *bcmf26a/b* plants. (A–H) Transgenic plants obtained. (A) After growth for 4 d to 5 d, the seedlings were obtained. (B) The cotyledon with petiole was cut from the seedling for pre-culture in MS media for 2 d to 3 d. (C) The explants were co-cultured with *A*. *tumefaciens* containing the *BcMF26a/b* amiRNA construct for 2 d. (D) The explants were differentiating cultured in differentiating media containing hygromycin for two weeks. (E–F) The obtained resistant adventitious buds were sub-cultured and induced rooting. (G–H) Putative transgenic plants of the *bcmf26a/b* and control lines were transplanted into the pot. (I–T) Morphological observation of the *bcmf26a/b* and control transgenic plants. No distinct differences were observed during the vegetative growth phases and flower organs between the (I, K, M, O, Q, and S) *bcmf26a/b* transgenic plants and (J, L, N, P, R, and T) control plants.
(TIF)

S1 Table The primer names, primer sequences, and annealing temperatures (Tm) for sequence amplification and transcript verification. (DOC)

S2 Table Details of the PG genes used in multiple alignment and phylogenetic analysis. (DOC)

## References

[1] Taylor LP, Hepler PK. Pollen germination and tube growth. Annu Rev Plant Physiol. 1997; 48: 461–491. 10.1146/annurev.arplant.48.1.461 15012271

[2] Ariizumi T, Toriyama K. Genetic regulation of sporopollenin synthesis and pollen exine development. Annu Rev Plant Biol. 2011; 62: 437–460. 10.1146/annurev-arplant-042809-112312 21275644

[3] Owen HA, Makaroff CA. Ultrastructure of microsporogenesis and microgametogenesis in *Arabidopsis thaliana* (L.) Heynh. ecotype Wassilewskija (*Brassicaceae*). Protoplasma 1995; 185: 7–21.

[4] Dick-Perez M, Wang T, Salazar A, Zabotina OA, Hong M. Multidimensional solid-state NMR studies of the structure and dynamics of pectic polysaccharides in uniformly C-13-labeled *Arabidopsis* primary cell walls. Magn Reson Chem. 2012; 50: 539–550. 10.1002/mrc.3836 22777793

[5] Caffall KH, Mohnen D. The structure, function, and biosynthesis of plant cell wall pectic polysaccharides. Carbohyd Res 2009; 344: 1879–1900. 10.1016/j.carres.2009.05.021 19616198

[6] Hadfield KA, Bennett AB. Polygalacturonases: Many genes in search of a function. Plant Physiol. 1998; 117: 337–343. 9625687 10.1104/pp.117.2.337 PMC1539180

[7] Demura T, Tashiro G, Horiguchi G, Kishimoto N, Kubo M, Matsuoka N, et al Visualization by comprehensive microarray analysis of gene expression programs during transdifferentiation of mesophyll cells into xylem cells. P Natl Acad Sci USA. 2002; 99: 15794–15799. 10.1073/pnas.232590499 PMC137795 12438691

[8] Sander L, Child R, Ulvskov P, Albrechtsen M, Borkhardt B. Analysis of a dehiscence zone endo-polygalacturonase in oilseed rape (*Brassica napus*) and *Arabidopsis thaliana*: evidence for roles in cell separation in dehiscence and abscission zones, and in stylar tissues during pollen tube growth. Plant Mol Biol Rep. 2001; 46: 469–479. 10.1023/a:1010619002833 11485203

[9] Ogawa M, Kay P, Wilson S, Swain SM. *ARABIDOPSIS DEHISCENCE ZONE POLYGALACTURONASE1* (*ADPG1*), *ADPG2*, and *QUARTET2* are polygalacturonases required for cell separation during reproductive development in *Arabidopsis* . Plant Cell 2009; 21: 216–233. 10.1105/tpc.108.063768 19168715 PMC2648098

[10] Kim J, Shiu SH, Thoma S, Li WH, Patterson SE. Patterns of expansion and expression divergence in the plant polygalacturonase gene family. Genome Biol. 2006; 7: R87 17010199 10.1186/gb-2006-7-9-r87 PMC1794546

[11] Rhee SY, Somerville CR. Tetrad pollen formation in quartet mutants of *Arabidopsis thaliana* is associated with persistence of pectic polysaccharides of the pollen mother cell wall. Plant J. 1998; 15: 79–88. 9744097 10.1046/j.1365-313x.1998.00183.x

[12] Rhee SY, Sborne EO, Poindexter PD, Somerville CR. Microspore separation in the *quartet 3* mutants of *Arabidopsis* is impaired by a defect in a developmentally regulated polygalacturonase required for pollen mother cell wall degradation. Plant Physiol. 2003; 133: 1170–1180. 14551328 10.1104/pp.103.028266 PMC281612

[13] Huang L, Cao JS, Zhang AH, Ye YQ, Zhang YC, Liu TT. The polygalacturonase gene *BcMF2* from *Brassica campestris* is associated with intine development. J Exp Bot. 2009; 60: 301–313. 10.1093/jxb/ern295 19039102 PMC3071776

[14] Huang L, Ye Y, Zhang Y, Zhang A, Liu T, Cao J. *BcMF9*, a novel polygalacturonase gene, is required for both *Brassica campestris* intine and exine formation. Ann Bot. 2009; 104: 1339–1351. 10.1093/aob/mcp244 19815569 PMC2778392

[15] Zhang Q, Huang L, Liu TT, Yu XL, Cao JS. Functional analysis of a pollen-expressed polygalacturonase gene *BcMF6* in *Chinese cabbage* (*Brassica campestris* L. ssp. *chinensis* Makino). Plant Cell Rep. 2008; 27: 1207–1215. 10.1007/s00299-008-0541-x 18415101

[16] Zhang A, Qiu L, Huang L, Yu X, Lu G, Cao J. Isolation and characterization of an anther-specific polygalacturonase gene, *BcMF16*, in *Brassica campestris* ssp. *chinensis* . Plant Mol Biol Rep. 2012; 30: 330–338.

[17] Zhang A, Chen Q, Huang L, Qiu L, Cao J. Cloning and expression of an anther-abundant polygalacturonase gene *BcMF17* from *Brassica campestris* ssp. *chinensis* . Plant Mol Biol Rep. 2011; 29: 943–951.

[18] Carvajal F, Garrido D, Jamilena M, Rosales R. Cloning and characterisation of a putative pollen-specific polygalacturonase gene (*CpPG1*) differentially regulated during pollen development in zucchini (*Cucurbita pepo* L.). Plant Biol. 2014; 16: 457–466. 10.1111/plb.12070 23879260

[19] Gonzalez-Carranza ZH, Elliott KA, Roberts JA. Expression of polygalacturonases and evidence to support their role during cell separation processes in *Arabidopsis thaliana* . J Exp Bot. 2007; 58: 3719–3730. 17928369 10.1093/jxb/erm222

[20] Yang ZL, Liu HJ, Wang XR, Zeng QY. Molecular evolution and expression divergence of the *Populus* polygalacturonase supergene family shed light on the evolution of increasingly complex organs in plants. New Phytol. 2013; 197: 1353–1365. 10.1111/nph.12107 23346984

[21] Cao J. The Pectin Lyases in *Arabidopsis thaliana*: evolution, selection and expression profiles. Plos One 2012; 7: e46944 10.1371/journal.pone.0046944 23056537 PMC3467278

[22] Park KC, Kwon SJ, Kim NS. Intron loss mediated structural dynamics and functional differentiation of the polygalacturonase gene family in land plants. Genes Genom. 2010; 32: 570–577.

[23] Park KC, Kwon SJ, Kim PH, Bureau T, Kim NS. Gene structure dynamics and divergence of the polygalacturonase gene family of plants and fungus. Genome 2008; 51; 30–40. 10.1139/g07-093 18356937

[24] Blanc G, Hokamp K, Wolfe KH. A recent polyploidy superimposed on older large-scale duplications in the *Arabidopsis* genome. Genome Res. 2003; 13: 137–144. 12566392 10.1101/gr.751803 PMC420368

[25] Simillion C, Vandepoele K, Van Montagu MCE, Zabeau M, Van de Peer Y. The hidden duplication past of *Arabidopsis thaliana* . P Natl Acad Sci USA. 2002; 99: 13627–13632. 10.1073/pnas.212522399 PMC129725 12374856

[26] Torki M, Mandaron P, Mache R, Falconet D. Characterization of a ubiquitous expressed gene family encoding polygalacturonase in *Arabidopsis thaliana* . Gene 2000; 242: 427–436. 10721737 10.1016/s0378-1119(99)00497-7

[27] Huang L, Zhao XF, Liu TT, Dong H, Cao JS. Developmental characteristics of floral organs and pollen of *Chinese cabbage* (*Brassica campestris* L. ssp *chinensis*). Plant Syst Evol. 2010; 286: 103–115.

[28] Allen GC, Flores-Vergara MA, Krasnyanski S, Kumar S, Thompson WF. A modified protocol for rapid DNA isolation from plant tissues using cetyltrimethylammonium bromide. Nat Protoc. 2006; 1: 2320–2325. 17406474 10.1038/nprot.2006.384

[29] Livak KJ, Schmittgen TD. Analysis of relative gene expression data using real-time quantitative PCR and the 2(T)(-Delta Delta C) method. Methods 2001; 25: 402–408. 11846609 10.1006/meth.2001.1262

[30] Karimi M, Inze D, Depicker A. GATEWAY((TM)) vectors for *Agrobacterium*-mediated plant transformation. Trends Plant Sci. 2002; 7: 193–195. 11992820 10.1016/s1360-1385(02)02251-3

[31] Gan C. Gene gun accelerates DNA-coated particles to transform intact-cells. Scientist 1989; 3: 25–25.

[32] Clough SJ, Bent AF. Floral dip: a simplified method for *Agrobacterium*-mediated transformation of *Arabidopsis thaliana* . Plant J. 1998; 16: 735–743. 10069079 10.1046/j.1365-313x.1998.00343.x

[33] Sun L, van Nocker S. Analysis of promoter activity of members of the *PECTATE LYASE-LIKE* (*PLL*) gene family in cell separation in *Arabidopsis* . Bmc Plant Biol. 2010; 10: 152 10.1186/1471-2229-10-152 20649977 PMC3017822

[34] Schwab R, Ossowski S, Riester M, Warthmann N, Weigel D. Highly specific gene silencing by artificial microRNAs in *Arabidopsis* . Plant Cell 2006; 18: 1121–1133. 16531494 10.1105/tpc.105.039834 PMC1456875

[35] Yu XL, Cao JS, Ye WZ, Wang YQ. Construction of an antisense *CYP86MF* gene plasmid vector and production of a male-sterile *Chinese cabbage* transformant by the pollen-tube method. J Hortic Sci Biotech. 2004; 79: 833–839.

[36] Alexander MP. Differential staining of aborted and nonaborted pollen. Stain Technology 1969; 44: 117–122. 4181665 10.3109/10520296909063335

[37] Jiang J, Yao L, Yu Y, Lv M, Miao Y, Cao J. *PECTATE LYASE-LIKE10* is associated with pollen wall development in *Brassica campestris* . J Integr Plant Biol. 2014; 56: 1095–1105. 10.1111/jipb.12209 24773757

[38] Huang L, Cao J, Ye W, Liu T, Jiang L, Ye Y. Transcriptional differences between the male-sterile mutant *bcms* and wild-type *Brassica campestris* ssp *chinensis* reveal genes related to pollen development. Plant Biol. 2008; 10: 342–355. 10.1111/j.1438-8677.2008.00039.x 18426481

[39] Markovic O, Janecek S. Pectin degrading glycoside hydrolases of family 28: sequence-structural features, specificities and evolution. Protein Eng. 2001; 14: 615–631. 11707607 10.1093/protein/14.9.615

[40] Yu YJ, Lv ML, Liang Y, Xiong XP, Cao JS. Molecular cloning and characterization of a novel polygalacturonase gene, *BcMF24*, involved in pollen development of *Brassica campestris* ssp. *chinensis* . Plant Mol Biol Rep. 2014; 32: 476–486.

[41] Maere S, De Bodt S, Raes J, Casneuf T, Van Montagu M, Kuiper M, et al Modeling gene and genome duplications in eukaryotes. P Natl Acad Sci USA. 2005; 102: 5454–5459. 10.1073/pnas.0501102102 PMC556253 15800040

[42] Freeling M. Bias in plant gene content following different sorts of duplication: Tandem, Whole-Genome, Segmental, or by Transposition. Annu Rev Plant Biol. 2009; 60: 433–453. 10.1146/annurev.arplant.043008.092122 19575588

[43] Bowers JE, Chapman BA, Rong JK, Paterson AH. Unravelling angiosperm genome evolution by phylogenetic analysis of chromosomal duplication events. Nature 2003; 422: 433–438. 12660784 10.1038/nature01521

[44] Xu GX, Guo CC, Shan HY, Kong HZ. Divergence of duplicate genes in exon-intron structure. P Natl Acad Sci USA. 2012; 109: 1187–1192. 10.1073/pnas.1109047109 PMC3268293 22232673

[45] Blanc G, Wolfe KH. Functional divergence of duplicated genes formed by polyploidy during *Arabidopsis* evolution. Plant Cell 2004; 16: 1679–1691. 15208398 10.1105/tpc.021410 PMC514153

[46] Saleh A, Alvarez-Venegas R, Yilmaz M, Le O, Hou GC, Sadder M, et al The highly similar *Arabidopsis* homologs of trithorax *ATX1* and *ATX2* encode proteins with divergent biochemical functions. Plant Cell 2008; 20: 568–579. 10.1105/tpc.107.056614 18375658 PMC2329920

[47] Duarte JM, Cui LY, Wall PK, Zhang Q, Zhang XH, Leebens-Mack J, et al Expression pattern shifts following duplication indicative of subfunctionalization and neofunctionalization in regulatory genes of *Arabidopsis* . Mol Biol Evol. 2006; 23: 469–478. 16280546 10.1093/molbev/msj051

[48] Gu X. Evolution of duplicate genes versus genetic robustness against null mutations. Trends Genet. 2003;19: 354–356. 12850437 10.1016/S0168-9525(03)00139-2

[49] Danilevskaya ON, Hermon P, Hantke S, Muszynski MG, Kollipara K, Ananiev EV. Duplicated *fie* genes in maize: expression pattern and imprinting suggest distinct functions. Plant Cell 2003; 15: 425–438. 12566582 10.1105/tpc.006759 PMC141211

[50] Qi MF, Xu T, Chen WZ, Li TL. Ultrastructural localization of polygalacturonase in ethylene-stimulated abscission of Tomato pedicel explants. Scientific World J. 2014;105:1–9. 10.1155/2014/389896 PMC3982476 24790564

[51] Nickel W. Unconventional secretory routes: direct protein export across the plasma membrane of mammalian cells. Traffic. 2005; 6: 607–614. 15998317 10.1111/j.1600-0854.2005.00302.x

[52] Nickel W. The mystery of nonclassical protein secretion—a current view on cargo proteins and potential export routes. Eur J Biochem. 2003; 270: 2109–2119. 12752430 10.1046/j.1432-1033.2003.03577.x

[53] Lynch M, Conery JS. The evolutionary fate and consequences of duplicate genes. Science 2000; 290: 1151–1155. 11073452 10.1126/science.290.5494.1151

[54] Wang XW, Wang HZ, Wang J, Sun RF, Wu J, Liu SY, et al The genome of the mesopolyploid crop species *Brassica rapa* . Nat Genet. 2011; 43: 1035–1039. 10.1038/ng.919 21873998

[55] Kirik V, Simon M, Wester K, Schiefelbein J, Hulskamp M. Enhancer of *TRY* and *CPC 2* (*ETC2*) reveals redundancy in the region-specific control of trichome development of *Arabidopsis* . Plant Mol Biol. 2004; 55: 389–398. 15604688 10.1007/s11103-004-0893-8

[56] Honys D, Twell D. Comparative analysis of the *Arabidopsis* pollen transcriptome. Plant Physiol. 2003; 132: 640–652. 12805594 10.1104/pp.103.020925 PMC167004

[57] Li S, Ge FR, Xu M, Zhao XY, Huang GQ, Zhou LZ, et al *Arabidopsis* COBRA-LIKE 10, a GPI-anchored protein, mediates directional growth of pollen tubes. Plant J. 2013; 74: 486–497. 10.1111/tpj.12139 23384085

[58] Li YQ, Chen F, Linskens HF, Cresti M. Distribution of unesterified and esterified pectins in cell-walls of pollen tubes of flowering plants. Sex Plant Reprod. 1994; 7: 145–152.

[59] Castro AJ, Suarez C, Zienkiewicz K, de Dios Alche J, Zienkiewicz A, Isabel Rodriguez-Garcia M. Electrophoretic profiling and immunocytochemical detection of pectins and arabinogalactan proteins in olive pollen during germination and pollen tube growth. Ann Bot. 2013; 112: 503–513. 10.1093/aob/mct118 23712452 PMC3718210

[60] Yeung EC, Oinam GS, Yeung SS, Harry I. Anther, pollen and tapetum development in safflower, *Carthamus tinctorius* L. Sex Plant Reprod. 2011; 24: 307–317. 10.1007/s00497-011-0168-x 21573927

[61] Moon S, Kim SR, Zhao GC, Yi J, Yoo Y, Jin P, et al Rice *GLYCOSYLTRANSFERASE1* encodes a glycosyltransferase essential for pollen wall formation. Plant Physiol. 2013; 161: 663–675. 10.1104/pp.112.210948 23263792 PMC3561011

[62] Ueda K, Yoshimura F, Miyao A, Hirochika H, Nonomura KI, Wabiko H. *COLLAPSED ABNORMAL POLLEN1* gene encoding the arabinokinase-like protein is involved in pollen development in rice. Plant Physiol. 2013; 162: 858–871. 10.1104/pp.113.216523 23629836 PMC3668075

[63] Heslopharrison J. Pollen germination and pollen-tube growth. Int Rev Cytol. 1987; 107: 1–78.

[64] Geitmann A. How to shape a cylinder: pollen tube as a model system for the generation of complex cellular geometry. Sex Plant Reprod. 2010; 23: 63–71. 10.1007/s00497-009-0121-4 20165964

[65] Kim J, Patterson S. Expression divergence and functional redundancy of polygalacturonases in floral organ abscission. Plant Signal Behav. 2006; 1: 281–283. 19704626 10.4161/psb.1.6.3541 PMC2634239

