# Supplementary figures and images for: BcMF26a and BcMF26b Are Duplicated Polygalacturonase Genes with Divergent Expression Patterns and Functions in Pollen Development and Pollen Tube Formation in Brassica campestris
Source: PLoS One. 2015 Jul 8;10(7):e0131173. doi: 10.1371/journal.pone.0131173 (PMC4495986; doi:10.1371/journal.pone.0131173)

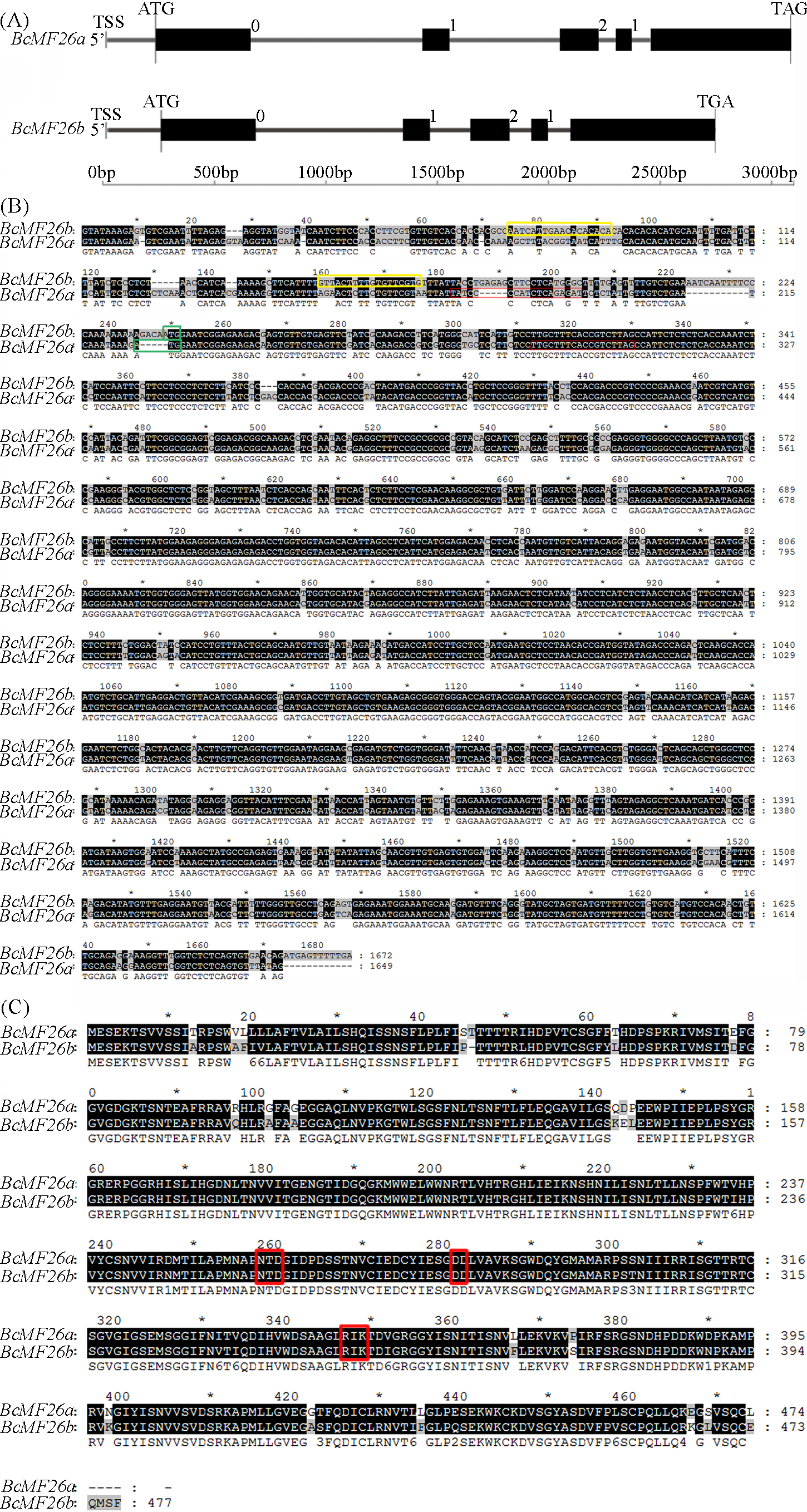

Supplement: S1 Fig — (A) The positions of exons and introns are shown. Both of them contain five exons (black boxes) and four introns (thin lines). The numbers above indicate the intron phases. (B) Nucleotide sequence alignment of BcMF26a and BcMF26b. The sequences of the two genes share high similarity. The positions of the primers used for qRT-PCR are shown for BcMF26a and BcMF26b with red boxes and yellow boxes, respectively. The locations of the start codon are marked by green boxes. (C) Amino acid sequence alignment of BcMF26a and BcMF26b. The amino acid sequences of the two genes have high similarity. The locations of the four conserved motifs of PG protein are marked by red boxes. BcMF26a, BcMF26b, and At4g33440 all contained three out of the four typical conserved domains. (TIF) [file pone.0131173.s001.tif]

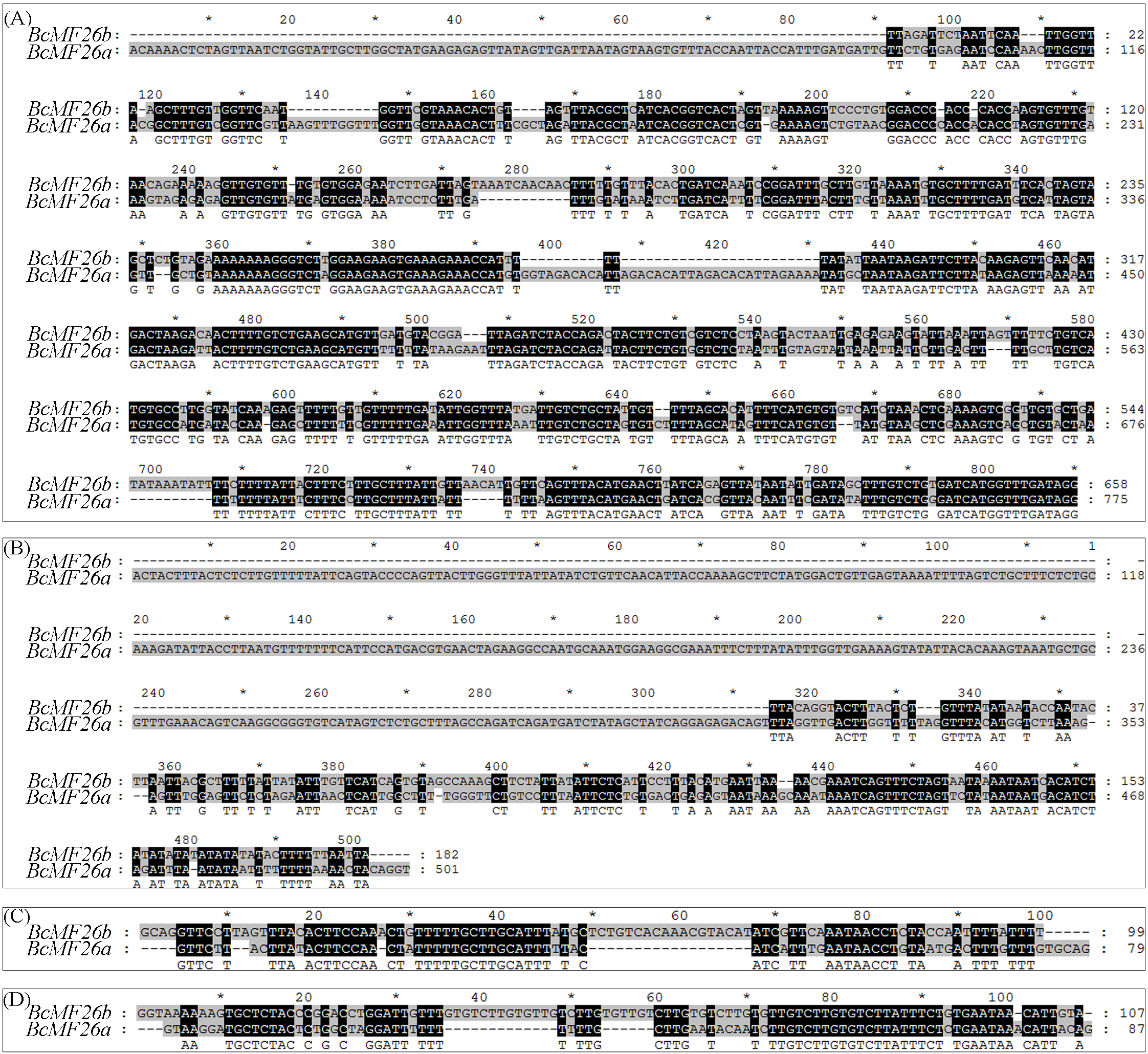

Supplement: S2 Fig — (A–D) Alignment results of the intron sequences between BcMF26a and BcMF26b by ClustalX software. The lengths and sequence similarities of the four pairs of introns have large divergences. (TIF) [file pone.0131173.s002.tif]

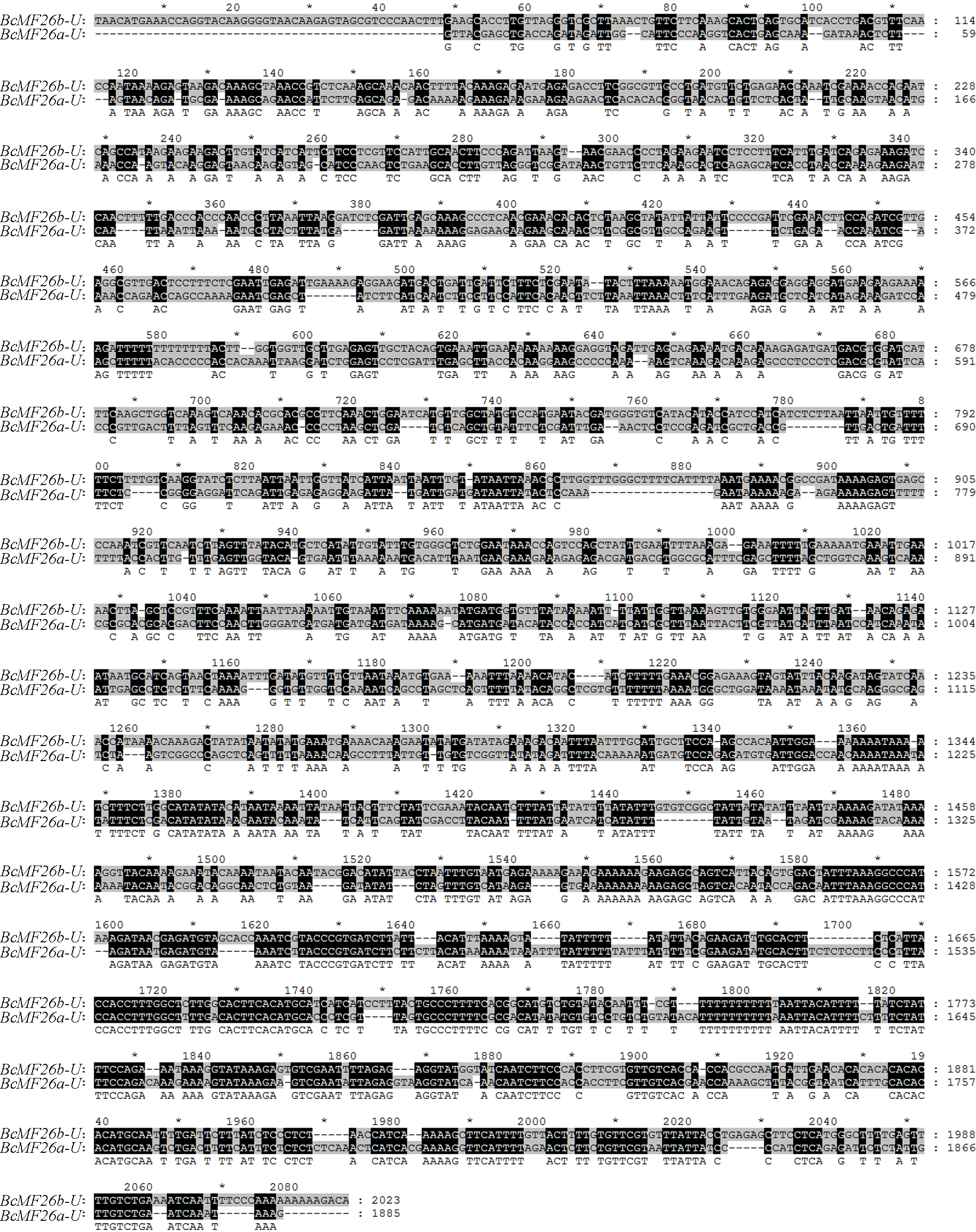

Supplement: S3 Fig — The significant divergences are presented. BcMF26a-U and BcMF26b-U indicate the names of deduced regulatory sequences upstream of ‘ATG’. (TIF) [file pone.0131173.s003.tif]

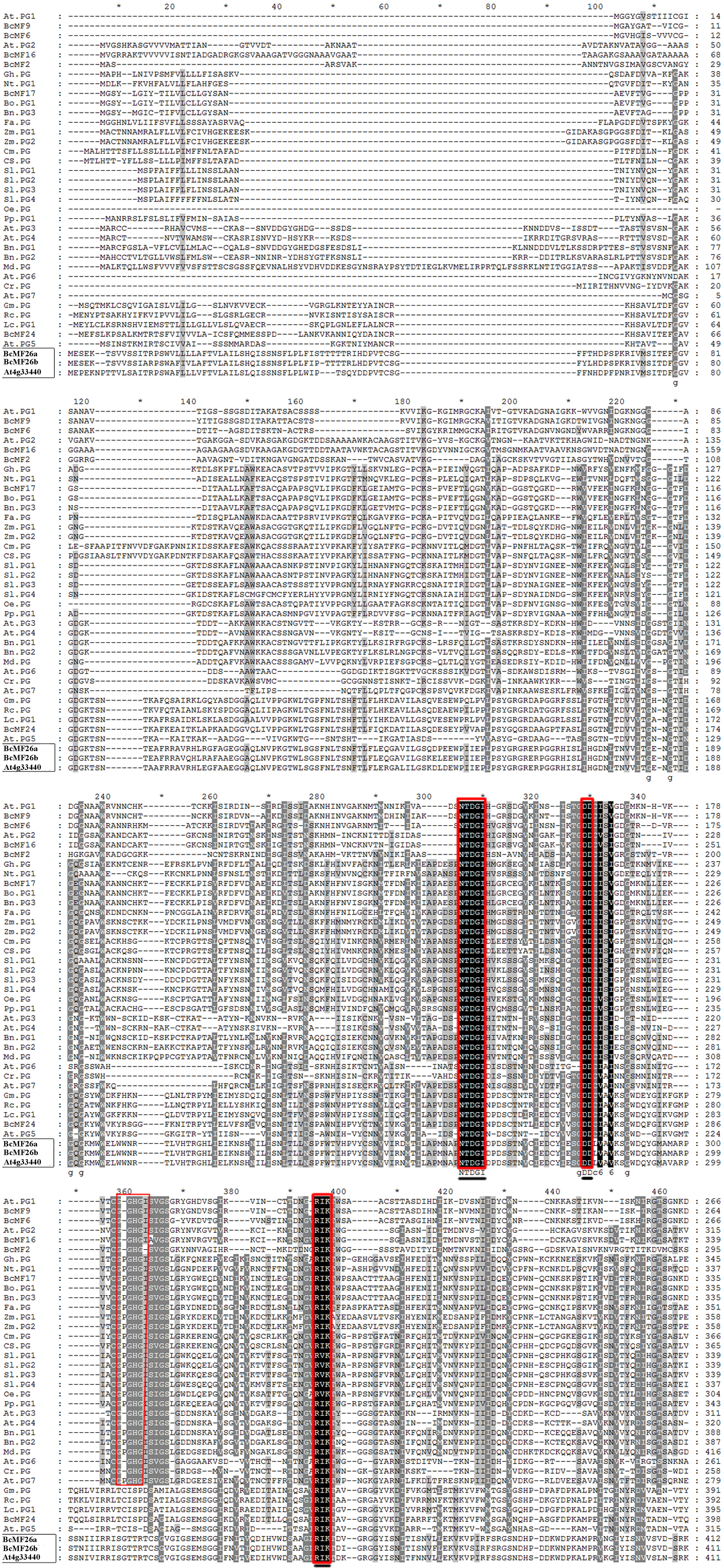

Supplement: S4 Fig — BcMF26a, BcMF26b, and At4g33440 contain three out of the four typical conserved domains of PG protein. (TIF) [file pone.0131173.s004.tif]

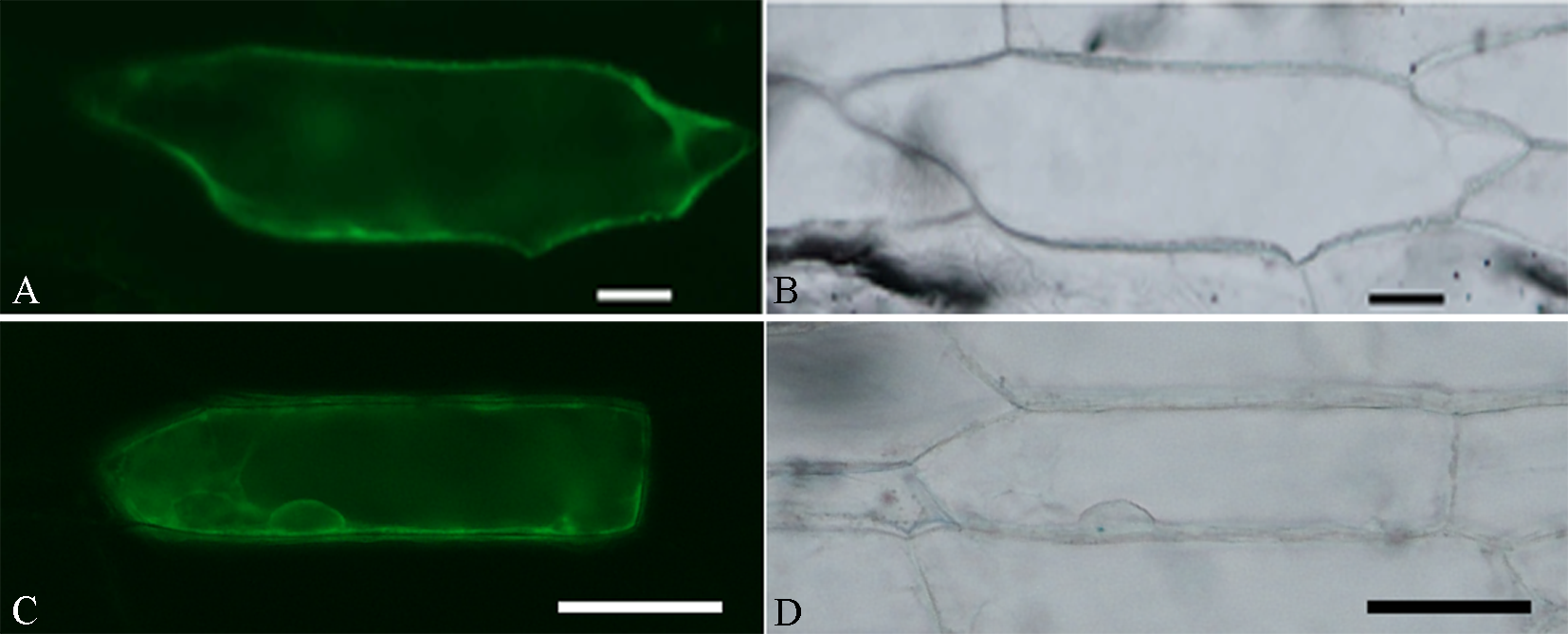

Supplement: S5 Fig — (A and C) GFP signal in the onion epidermal cells transformed with pBGWFS7.0–proBcMF26a: GUS-GFP and pBGWFS7.0–proBcMF26b: GUS-GFP vectors, respectively. (B and D) Bright field images of the corresponding onion epidermal cells. Scale bars = 50 μm. (TIF) [file pone.0131173.s005.tif]

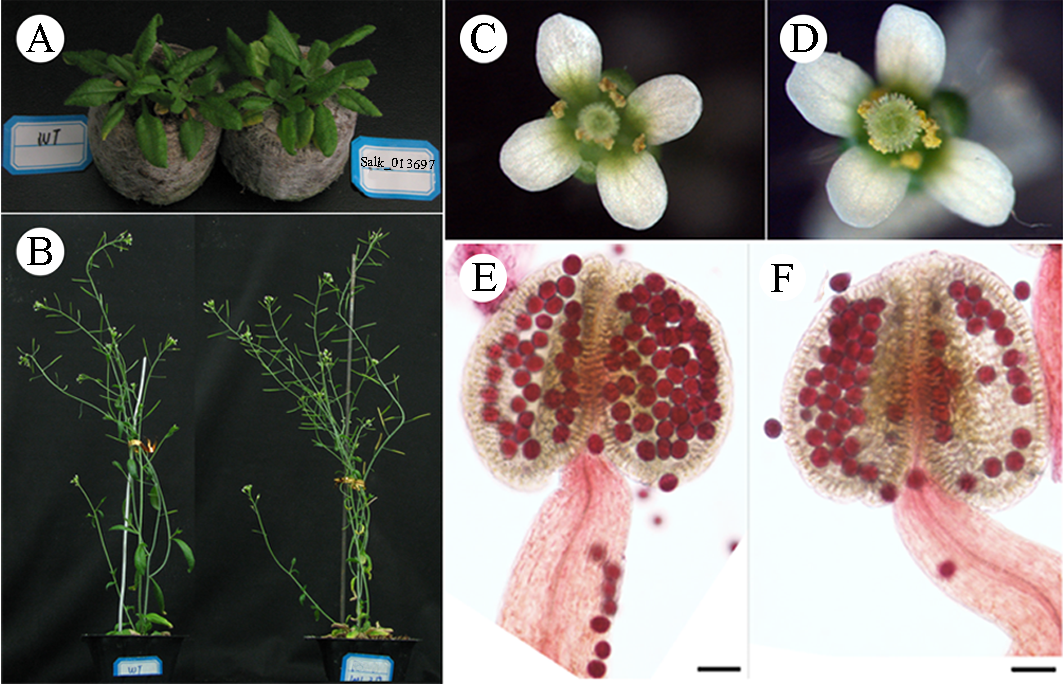

Supplement: S6 Fig — (A) Growth status of the wild–type and SALK_013697 plants at the rosette leaf stage, respectively. (B) Growth status of 35-day-old plants of the wild–type plants and SALK_013697 mutants, respectively. (C and D) Growth status of the flowers of the wild–type plants and SALK_013697 mutants, respectively. (E and F) Alexander staining of pollen grains of the wild type plants and SALK_013697 mutants, respectively. No obvious difference was observed between the mutant and the wild type. (TIF) [file pone.0131173.s006.tif]

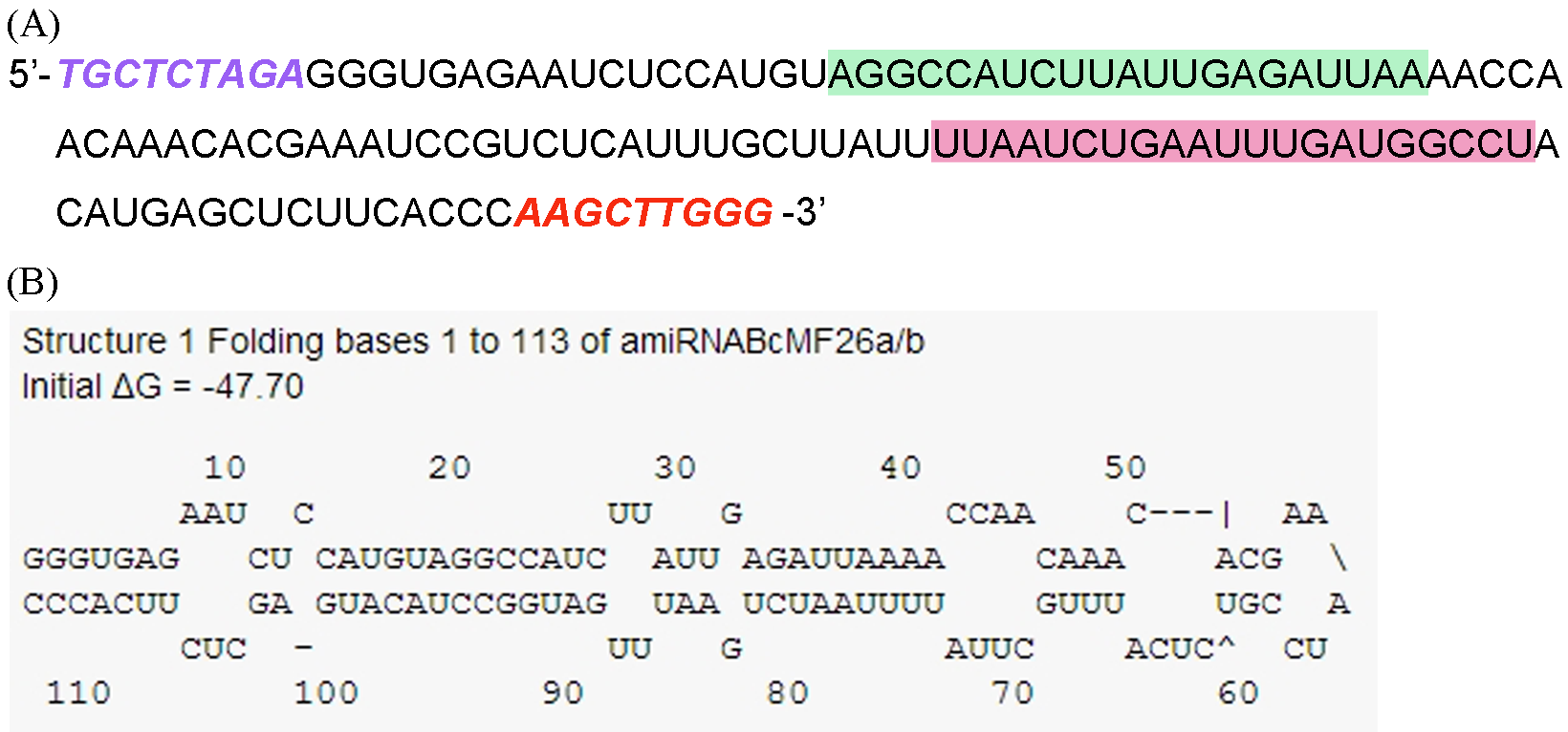

Supplement: S7 Fig — (A) Sequence used for constructing the amiRNA. The amiRNA was constructed based on the structure of MIR164a. The bases marked with green shadow indicate the selected sequence for constructing the amiRNA of BcMF26a/b; the bases marked with red shadow indicate its reverse complemented sequence. The blue and red bases indicate the restriction enzyme sites XbaI and HindIII, respectively. (B) The RNA folding form of the amiRNA, which was predicted by the mfold Web Server. (TIF) [file pone.0131173.s007.tif]

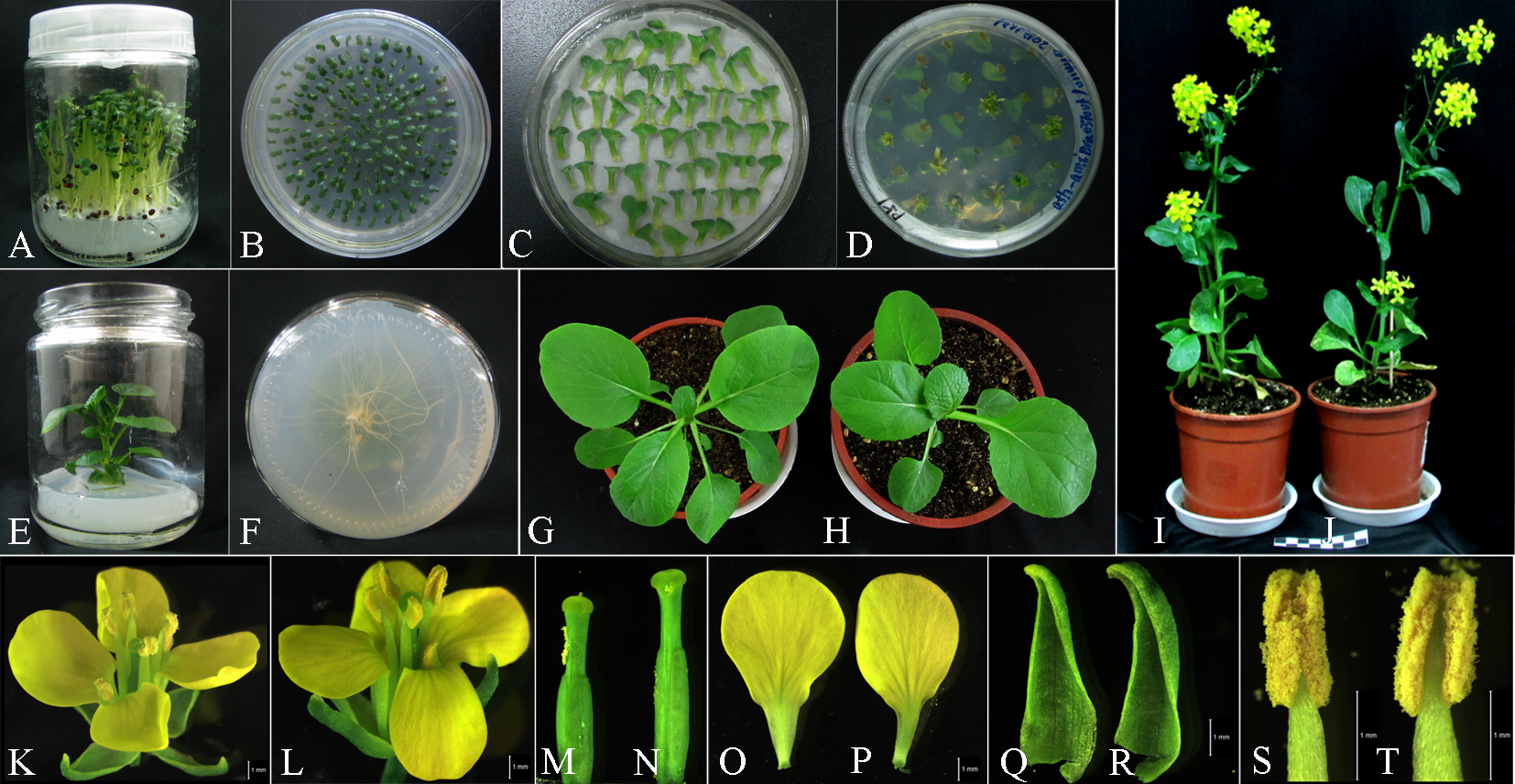

Supplement: S8 Fig — (A–H) Transgenic plants obtained. (A) After growth for 4 d to 5 d, the seedlings were obtained. (B) The cotyledon with petiole was cut from the seedling for pre-culture in MS media for 2 d to 3 d. (C) The explants were co-cultured with A. tumefaciens containing the BcMF26a/b amiRNA construct for 2 d. (D) The explants were differentiating cultured in differentiating media containing hygromycin for two weeks. (E–F) The obtained resistant adventitious buds were sub-cultured and induced rooting. (G–H) Putative transgenic plants of the bcmf26a/b and control lines were transplanted into the pot. (I–T) Morphological observation of the bcmf26a/b and control transgenic plants. No distinct differences were observed during the vegetative growth phases and flower organs between the (I, K, M, O, Q, and S) bcmf26a/b transgenic plants and (J, L, N, P, R, and T) control plants. (TIF) [file pone.0131173.s008.tif]
