# Supplementary material for: BcMF26a and BcMF26b Are Duplicated Polygalacturonase Genes with Divergent Expression Patterns and Functions in Pollen Development and Pollen Tube Formation in Brassica campestris
Source: PLoS One. 2015 Jul 8;10(7):e0131173. doi: 10.1371/journal.pone.0131173 (PMC4495986; doi:10.1371/journal.pone.0131173)
Supplement: S1 Table — (DOC) [file pone.0131173.s009.doc]

| **S1 Table The primer names, primer sequences and annealing temperatures (Tm) for sequence amplification and transcript verification.** | | | |
| --- | --- | --- | --- |
| **Primer name** | | **Primer sequence** | **Tm** |
| **Primer 1** | *BcMF26a*-DNA-UP-1 | AGATGGAATCGGAGAAGAC | 55°C |
|  | *BcMF26a*-DNA-DP-1 | AGACTATGACACCCGCCTTGA |  |
|  | *BcMF26a*-DNA-UP-2 | CTCGAAAGTCAGCTGTAC | 54°C |
|  | *BcMF26a*-DNA-DP-2 | ACTCATGGACCTCCTATA |  |
| **Primer 2** | *BcMF26a*-ORF-UP | ATGGAATCGGAGAAGACAAG | 54°C |
|  | *BcMF26a*-ORF-DP | CTATAAACACTGAGAGACCGAAGC |  |
| **Primer 3** | *BcMF26b*-DNA-UP-1 | TTCGTGTTTATTACCTGAG | 53°C |
|  | *BcMF26b*-DNA-DP-1 | CGATATGTACGTTTGTGAC |  |
|  | *BcMF26b*-DNA-UP-2 | GTGCATACTAGAGGCCATC | 55°C |
|  | *BcMF26b*-DNA-DP-2 | ACCAAAACACCGTTCAATC |  |
| **Primer 4** | *BcMF26b*-ORF-UP | ATGGAATCGGAGAAGACGAG | 54°C |
|  | *BcMF26b*-ORF-DP | TCAAAAACTCATCTGTTCACACTG |  |
| **Primer 5** | *BcMF26a*-QRT-PCR-UP | TATCCCCATCTCAGAGAT | 54°C |
|  | *BcMF26a*-QRT-PCR-DP | CTAAGACGGTGAAAGCAA |  |
| **Primer 6** | *BcMF26b*-QRT-PCR-UP | AATCATTGAACACACACA | 54°C |
|  | *BcMF26b*-QRT-PCR-DP | CACGAACACAAAAGTAAC |  |
| **Primer 7** | *UBC-10-*UP | GGGTCCTACAGACAGTCCTTAC | 54°C |
|  | *UBC-10-*DP | ATGGAACACCTTCGTCCTAAA |  |
| **Primer 8** | *BcMF26a*-promoter-UP | CACCGTTACGAGCTGACCAGATAGA | 66°C |
|  | *BcMF26a*-promoter-DP | AGGGAGGAATGAATTGGAGGAGAT |  |
| **Primer 9** | *BcMF26b*-promoter-UP | CACCTAACATGAAACCAGGTACA | 58°C |
|  | *BcMF26b*-promoter-DP | GATAGAACTCACAACACTCGTC |  |
| **Primer 10** | *BcMF26a*-Subcellular localization- UP | *TGCTCTAGA*ATGGAATCGGAGAAGACAAGT | 56°C |
|  | *BcMF26a*-Subcellular localization- DP | *TCCCCCGGG*CTATAAACACTGAGAGACCGA |  |
| **Primer 11** | *BcMF26b*-Subcellular localization- UP | *TGCTCTAGA*ATGGAATCGGAGAAGACGAG | 56°C |
|  | *BcMF26b*-Subcellular localization- DP | *TCCCCCGGG*TCAAAAACTCATCTGTTCAC |  |
| **Primer 12** | SALK_013967*-*T-DNA-UP | CTGACGAGATTGTAACGCCTC | 59°C |
|  | SALK_013967*-*T-DNA-DP | AATGGTGGGTCCATTTTAACC |  |
|  | LBb1.3 | ATTTTGCCGATTTCGGAAC |  |
| **Primer 13** | SALK_028430*-*T-DNA-UP | GTCATTACAGGAGAAAA | 58°C |
|  | SALK_028430*-*T-DNA-DP | ACAAGATCATCACCACT |  |
|  | LBb1.3 | ATTTTGCCGATTTCGGAAC |  |
| **Primer 14** | *At4g33440-*RT-PCR-UP | TCCAAGGACCTAGACGAAT | 54°C |
|  | *At4g33440-*RT-PCR-DP | CTCCATGAATGAGGCTGAT |  |
| **Primer 15** | *Tubulin*-UP | GACGCATGTCCATGAAGGAG |  |
|  | *Tubulin*-DP | CCAATGCAAGAAAGCCTTGC |  |
| **Primer 16** | *BcMF26a/b*-QRT-PCR-UP | CTATCCATCCTGTTTACTGC | 54°C |
|  | *BcMF26a/b*-QRT-PCR-DP | GTGCTTGAGTCTGGGTCTA |  |
