# Supplementary material for: BcMF26a and BcMF26b Are Duplicated Polygalacturonase Genes with Divergent Expression Patterns and Functions in Pollen Development and Pollen Tube Formation in Brassica campestris
Source: PLoS One. 2015 Jul 8;10(7):e0131173. doi: 10.1371/journal.pone.0131173 (PMC4495986; doi:10.1371/journal.pone.0131173)
Supplement: S2 Table — (DOC) [file pone.0131173.s010.doc]

| **S2 Table Details of the PG genes used in the multiple alignment and phylogenetic analysis.** | | | | |
| --- | --- | --- | --- | --- |
| **Abbreviation** | **Species (gene name)** | | **GenBank No. or reference** | |
| At.PG1 | *Arabidopsis thaliana* (*PGA3*) | NP_187439.1 | |  |
| At.PG2 | *Arabidopsis thaliana* (*PGA2*) | CAA51692.1 | |  |
| At.PG3 | *Arabidopsis thaliana* (*ADPG1*) | NP_191310.1 | |  |
| At.PG4 | *Arabidopsis thaliana* (*ADPG2*) | NP_850359.1 | |  |
| At.PG5 | *Arabidopsis thaliana* | ABE65854.1 | |  |
| At.PG6 | *Arabidopsis thaliana* | [CAA20037.1](http://www.ncbi.nlm.nih.gov/protein/3367585?report=genbank&log$=prottop&blast_rank=7&RID=3HR8MPBZ014) | |  |
| At.PG7 | *Arabidopsis thaliana* | [NM_123851](http://www.ncbi.nlm.nih.gov/entrez/viewer.cgi?db=nucleotide&val=NM_123851) | |  |
| At4g33440 | *Arabidopsis thaliana* | [NM_119498](http://www.ncbi.nlm.nih.gov/entrez/viewer.cgi?db=nucleotide&val=NM_119498) | |  |
| BcMF2 | *Brassica campestris* | ABW24665.1 | |  |
| BcMF6 | *Brassica campestris* | ACP74159.1 | |  |
| BcMF9 | *Brassica campestris* | ABN13878.1 | |  |
| BcMF16 | *Brassica campestris* | ADJ68232.1 | |  |
| BcMF17 | *Brassica campestris* | Zhang et al.(2012) | |  |
| BcMF24 | *Brassica campestris* | Yu et al.(2014) | |  |
| Bn.PG1 | *Brassica napus* (*RDPG*) | CAA65072.1 | |  |
| Bn.PG2 | *Brassica napus* (*PGAZ*) | CAC05658.1 | |  |
| Bn.PG3 | *Brassica napus* | [CDY22140.1](http://www.ncbi.nlm.nih.gov/protein/674910918?report=genbank&log$=prottop&blast_rank=35&RID=3FH51N8Y014) | |  |
| Bo.PG | *Brassica oleracea* | AAM74219.1 | |  |
| Cm.PG | *Cucumis melo* (*MPG2*) | AAC26511.1 | |  |
| Cr.PG | *Capsella rubella* | [XP_006279827.1](http://www.ncbi.nlm.nih.gov/protein/565430965?report=genbank&log$=prottop&blast_rank=22&RID=3HR8MPBZ014) | |  |
| Cs.PG | *Cucumis sativus* | [XP_004133718.1](http://www.ncbi.nlm.nih.gov/protein/449431860?report=genbank&log$=prottop&blast_rank=2&RID=3FMB5E5W01R) | |  |
| Fa.PG | *Fragaria ananassa* | ABE77145.1 | |  |
| Gh.PG | *Gossypium hirsutum* (*G9*) | AAA82167.1 | |  |
| Gm.PG | *Glycine max* | XP_003535598.1 | |  |
| Lc.PG1 | *Litchi chinensisv (LcPG1)* | AFW04075.1 | |  |
| Sl.PG1 | *Solanum lycopersicum* (*TAPG1*) | AAC28903.1 | |  |
| Sl.PG2 | *Solanum lycopersicum* (*TAPG2*) | AAC28904.1 | |  |
| Sl.PG3 | *Solanum lycopersicum* (*TAPG3*) | AAC28902.2 | |  |
| Sl.PG4 | *Solanum lycopersicum* (*TAPG4*) | AAB09576.1 | |  |
| Md.PG | *Malus domestica* | AAA74452.1 | |  |
| Nt.PG | *Nicotiana tabacum* (*PG1*) | Q05967.1 | |  |
| Oe.PG | *Olea europaea* | [ACA49228.1](http://www.ncbi.nlm.nih.gov/protein/169144941?report=genbank&log$=prottop&blast_rank=15&RID=3FMZ0T92015) | |  |
| Pp.PG | *Prunus persica* (*PRF5*) | CAA54150.1 | |  |
| Rc.PG | *Ricinus communis* | XP_002513653.1 | |  |
| Zm.PG1 | *Zea mays* | P26216.1 | |  |
| Zm.PG2 | *Zea mays* | [NP_001105432.1](http://www.ncbi.nlm.nih.gov/protein/162464324?report=genbank&log$=prottop&blast_rank=1&RID=3FNMV07Y014) | |  |
